# The spectral model of personal wellbeing and the validity of the Personal Wellbeing Spectral Questionnaire

**DOI:** 10.3389/fpsyg.2025.1719574

**Published:** 2026-01-15

**Authors:** Attila Oláh, Bella Bagdi, András Vargha

**Affiliations:** 1 Positive Psychology Research Group, Faculty of Education and Psychology, Institute of Psychology, Eötvös Loránd University, Budapest, Hungary; 2 Doctoral School of Psychology, Eötvös Loránd University, Budapest, Hungary; 3 Faculty of Humanities and Social Sciences, Institute of Psychology, Károli Gáspár Reformed Church University, Budapest, Hungary

**Keywords:** mental health, personal wellbeing, Personal Wellbeing Spectrum Questionnaire, second-order wellbeing factors, subjective wellbeing

## Abstract

**Introduction:**

Personal wellbeing is a multidimensional construct encompassing emotional, psychological, social, and spiritual aspects. To capture this complexity, we introduce the Personal Wellbeing Spectrum Theory, which organizes these dimensions into two higher-order domains: internal wellbeing (emotional and psychological) and external wellbeing (social and spiritual). The paper aimed to conceptualize this theory and to develop and validate the Personal Wellbeing Spectrum Questionnaire (PWBSQ), a tool designed to operationalize the model.

**Methods:**

The structural validity of PWBSQ was examined in a large online sample (*n* = 11,686) using confirmatory factor analysis, and the hierarchical model was tested in three additional independent samples. Reliability and measurement invariance across sociodemographic groups were assessed. Substantive validity was evaluated through associations with established measures, including the Positivity Scale, Diener Flourishing Scale, Huppert Flourishing Scale, Values in Action Inventory, Mental Health Test, and the PERMA model.

**Results:**

The hierarchical model demonstrated good fit across multiple samples (RMSEA around 0.06, SRMR round 0.04, CFI and TLI around 0.95), with consistent reliability (α and ω > 0.79). Measurement invariance was supported across five sociodemographic variables. The questionnaire showed meaningful associations with subjective financial situation, gender, education, and family characteristics, while marital status and occupation had only minor effects.

**Discussion:**

Findings confirm the Personal Wellbeing Spectrum Theory and support the PWBSQ as a reliable and valid instrument for assessing multidimensional wellbeing. This tool provides researchers and practitioners with a comprehensive measure of personal wellbeing that can be applied across diverse populations, facilitating both theoretical development and practical assessment in health and social sciences.

## 1 Introduction

Over the past four decades, research into wellbeing has been shaped by three major perspectives: the hedonic, the eudaimonic, and the integrative hedo-eudaimonic approach. Hedonic theories conceptualize wellbeing as maximizing pleasure and minimizing pain (Kahneman et al., 1999). Their roots lie both in classical philosophy such as Epicurus (341–270 BCE), and in modern psychology, notably Diener’s model of subjective wellbeing, which highlights positive affect and life satisfaction (Diener et al., 1985). In contrast, eudaimonic theories emphasize meaning, self-realization and the pursuit of the “good life,” drawing on Aristotle’s ethics (Gottlieb, 2009), Ryff’s model of psychological wellbeing (Ryff, 1989, 2021) and Keyes’ framework of social wellbeing (Keyes, 1998). Central to these models are autonomy, competence, and relatedness—core elements principles echoed in Deci and Ryan’s (2012) self-determination theory and Maslow’s (1968) hierarchy of self-actualization.

The integrative hedonic-eudaimonic paradigm, as presented in Seligman, Seligman’s (2011, 2018) PERMA model, combines pleasure, engagement, relationships, meaning and accomplishment to define wellbeing as flourishing. Diener et al.’s (2010) updated concept of flourishing further incorporates reflection, purpose, and achievement within the scope of holistic wellbeing. Recent meta-analyses and scoping reviews (e.g., Dodge et al., 2012; Linton et al., 2016; Zhang et al., 2024) have confirmed that global wellbeing comprises overlapping components, such as positive emotions, autonomy, meaningful relationships, self-fulfillment and purpose, which have been elaborated upon and empirically validated within these paradigms. Together, these findings suggest the possibility of a comprehensive, unifying framework that reflects the totality of human wellbeing.

Despite notable advances, existing measurement tools remain fragmented, often addressing wellbeing through isolated dimensions. Instruments such as Ryff’s Psychological WellBeing Scales (Ryff, 1989), the Personal Wellbeing Index (Cummins et al., 2003), the Warwick–Edinburgh Mental Wellbeing Scale (Tennant et al., 2007) have substantially enriched empirical research; however, they tend to conceptualize wellbeing as a set of discrete components rather than as an interdependent system.

Our Personal Wellbeing Spectrum Theory (PWBST) is a dynamic, integrative framework that builds on validated traditions of wellbeing research. It conceptualizes wellbeing as a spectrum encompassing emotional, psychological, social, and spiritual dimensions—core aspects of human functioning. Positive experiences in these domains represent the primary personal dimensions of wellbeing, reflecting the individual’s lived experience and subjective evaluation rather than societal indicators. The notion of a spectrum underscores the fluidity and interdependence of these dimensions, highlighting how their interactions foster personal flourishing. Wellbeing, in this view, emerges when individuals achieve emotional stability, psychological growth, meaningful social connections, and spiritual fulfillment, resulting in a balanced integration of inner and outer life. This approach offers a deeper understanding of the multidimensional nature of wellbeing and supports the design of targeted interventions to promote flourishing.

Recent empirical evidence underscores the multidimensional character of wellbeing: spiritual wellbeing has been shown to protect against burnout (Chirico et al., 2023), gratitude and self-esteem contribute to enhanced subjective wellbeing (Yildirim and Alshehri, 2019), and social inclusion fosters mental health among youth (Arslan et al., 2022). Additional studies highlight its context-sensitive nature, linking wellbeing to psychosocial indicators such as psychological capital (Momin and Rolla, 2024a), work–family balance (Momin and Rolla, 2024b), and organizational outcomes (Momin and Rolla, 2024c). Collectively, these findings emphasize the necessity of integrating emotional, psychological, social, and spiritual dimensions into a unified and coherent model.

The Personal Wellbeing Spectrum Questionnaire (PWBSQ) was developed to operationalize the PWBST by capturing emotional, psychological, social, and spiritual domains within a distinctive internal–external architecture of wellbeing. This structural feature, empirically validated in subsequent studies, has not been explicitly emphasized in previous models. Compared to flourishing measures such as those proposed by Diener et al.’s (2010) and Huppert and So (2013), which acknowledge multidimensionality but reduce it to single-scale operationalizations, the PWBSQ enables the identification of individual-specific patterns of wellbeing dimensions, supporting tailored interventions. In contrast to the PERMA model (Seligman, 2011) and PERMA Profiler (Butler and Kern, 2016). often regarded as gold standards, the PWBSQ extends the multidimensional spectrum by integrating the spiritual domain and distinguishing internal and external wellbeing—dimensions not explicitly emphasized in PERMA.

By synthesizing validated components from multiple paradigms into a unified framework, the PWBSQ provides a holistic and individualized measure of personal wellbeing, filling a critical gap in the measurement landscape and offering a foundation for research and intervention across diverse populations.

### 1.1 Present study

The present study aims to (a) present the Personal Wellbeing Spectrum Theory (PWBST), a multidimensional construct that integrates hedonic, eudaimonic, social-relational, and existential/spiritual paradigms; and (b) introduce the PWBSQ, a psychometric instrument designed to measure the psychosocial spectrum of personal wellbeing in accordance with this theory. The PWBSQ thus offers a comprehensive and person-centered instrument that enables nuanced assessment and supports evidence-based interventions across diverse contexts.

## 2 Materials and methods

### 2.1 The development of the Personal Wellbeing Questionnaire

To operationalize the PWBST, we developed the Personal Wellbeing Spectrum Questionnaire (PWBSQ) in the form of a short, 17-item questionnaire, which assesses the full range of personal wellbeing, including the four dimensions (emotional, psychological, social and spiritual) described above. The 17 items in the form of a 6-point Likert scale (see Supplementary Appendix) were the starting point for the definition of the four primary scales, representing the corresponding four dimensions based on their content (Oláh, 2021; Vargha et al., 2020). The four primary scales (*Emotional wellbeing* = *Emot*, *Psychological wellbeing* = *Psych*, *Social wellbeing* = *Soc*, *Spiritual wellbeing* = *Spirit*) of the PWBSQ measuring the emotional, psychological, social and spiritual components of personal wellbeing.

### 2.2 Research plan

Four studies were carried out to prepare and validate the final version of the PWBSQ. The aim of Study I was to finalize a set of items for which the multidimensional statistical model of personal wellbeing yields appropriate fit indices. The main aim of Studies II to IV was to confirm this model on new, independent samples. After this verification of structural validity, all four studies were used to check the substantive validity of the PWBSQ.

### 2.3 Presentation of the studies

#### 2.3.1 Study I

##### 2.3.1.1 Participants and procedure

Participants completed an 81-item online questionnaire. Ethical approval for the study was granted by the Research Ethics Committee of Eötvös Loránd University (permission number: 2019/21). Participation was voluntary and anonymous. Informed consent was obtained but no compensation was given. The valid study sample consisted of 11,686 persons (2,725 males and 8,961 females) in four subsamples. Two of them were investigated before the Covid outbreak in 2019 (NoCov19) and 2020 (NoCov20) and two during the Covid pandemic in 2021 (Cov21) and 2022 (Cov22) (see Table 1). The sociodemographic characteristics of the four subsamples are summarized in Tables 1, 2.

**TABLE 1:** Gender and age characteristics of the four subsamples of Study I.

Sample	*n*	Male	Female	Age mean	Age SD
1. NoCov19	4,459	954 (21.4%)	3,505 (78.6%)	43.8	15.5
2. NoCov20	3,024	839 (27.7%)	2,185 (72.3%)	49.7	14.7
3. Cov21	3,230	675 (20.9%)	2,555 (79.1%)	49.0	15.8
4. Cov22	973	257 (26.4%)	716 (73.6%)	36.8	10.9
Total	11,686	2,725 23.3%)	8,961 (76.7%)	46.2	15.6

**TABLE 2:** Sociodemographic characteristics of the participants of Study I (*n* = 11,686).

Age	18–25 years old: 12.4%	26–35 years old: 15.2%	36–50 years old: 31.2%	51–65 years old: 29.4%	66–100 years old: 11.7%
Education level	primary: 2.5%	secondary: 39.0%	college: 32.5%	university: 26.0%	
Number of children	0: 32.5%	1: 19.0%	2: 33.2%	3: 11.8%	3+: 3.4%
Type of settlement	Village: 22.6%	Small town: 19.0%	Middle town: 28.0%	Large town: 15.6%	Capital: 14.8%
Marital status	Lives alone: 16.0%	Cohabitation: 23.9%	Married: 45.3%	Widowed: 5.0%	Divorced: 9.8%
Occupation (other 2.8%)	Employee: 57.0%	Entrepreneur: 9.2%	Student: 8.0%	Pensioner: 19.3%	Unemployed: 3.6%
Subjective financial status	Poor: 2.2%	Below average: 9.4%	Average: 68.1%	Wealthy: 19.3%	Rich: 1.1%

Based on the data in Table 2, we can conclude that the sample was sufficiently heterogeneous for us to draw valid conclusions about the factor structure of PWBSQ. Although most participants in the sample were women (76.7%), the number of men (2,725) was also sufficiently large to ensure reliable results.

In terms of age, most of the participants (60.6%) were middle-aged (36–65 years), although the number of people under 26 (1450) and over 65 (1369) was also substantial. The sample was also balanced according to settlement type, since the number of respondents in all categories was over 1700. 96% of the participants had a high-school grade or higher. Regarding marital status, most of the participants were married (49.3%), while a small proportion (5.0%) were widowed. Most of the participants (57.0%) were employed, although there were also a significant proportion of pensioners (19.3%), students (8.0%), and entrepreneurs (9.2%). Most participants in the sample (68.1%) considered their financial situation to be average, although there were also a non-negligible proportion of wealthy people (19.3%). Only a small fraction of respondents declared themselves to be poor (2.2%) or rich (1.1%).

##### 2.3.1.2 Measures

Eight questions in the questionnaire referred to sociodemographic data (gender, age, education level, etc.) (see Tables 2, 3). One question (Positive experience%) assessed the percentage of the respondent’s recent positive experiences (1 = *10% positive experiences and 90% negative experiences*, …, 9 = *90% positive experiences and 10% negative experiences*). Four questions, scored using a six-point Likert scale, assessed the physical and psychological condition of the respondent: (1) Physical condition (My physical state is: 1 = *very bad*, 2 = *bad*, 3 = *acceptable*, 4 = *good*, 5 = *very good*, 6 = *excellent*); (2) General mental state (My general mental state is: 1 = *very bad*, 2 = *bad*, 3 = *acceptable*, 4 = *good*, 5 = *very good*, 6 = *excellent*); (3) General health condition (I am satisfied with my general health: 1 = *strongly disagree*, 2 = *moderately disagree*, 3 = *slightly disagree*, 4 = *slightly agree*, 5 = *moderately agree*, 6 = *strongly agree*); and (4) Physical strength (I feel strong and physically robust: 1 = *strongly disagree*, 2 = *moderately disagree*, 3 = *slightly disagree*, 4 = *slightly agree*, 5 = *moderately agree*, 6 = *strongly agree*). One question (Actual happiness) assessed the actual happiness of the respondent using seven numbered small faces (pictograms) side by side indicating decreasing levels of happiness with the following instruction: “Mark the number of the face that best expresses how happy you are at the moment” (scores: 1 = happiest face, 7 = least happy face). Prior to statistical analyses, the scale of this variable was inverted using a *y* = 8 − *x* transformation so that higher scores indicate higher levels of actual happiness.

**TABLE 3:** Results of first and second order four-factor CFA (rows 1–4) and first order one-factor CFA (row 5) obtained for PWBSQ.

CFA type	χ^2^	*df*	RMSEA	CI_90_(RMSEA)	pClose	CFI	TLI	SRMR
1st order	6262.29	112	0.069	(0.067; 0.070)	< 0.001	0.945	0.937	0.035
2nd order	6264.52	113	0.068	(0.067; 0.070)	< 0.001	0.945	0.937	0.035
2nd order –i15psy	4282.39	98	0.060	(0.059; 0.062)	< 0.001	0.960	0.951	0.032
1st order –i15psy	4283.05	97	0.061	(0.059; 0.062)	< 0.001	0.960	0.950	0.032
1st order –i15psy, 1 factor	15369.00	103	0.113	(0.111; 0.114)	< 0.001	0.853	0.828	0.065

In the table all χ^2^ values are significant at *p* < 0.001 level

###### 2.3.1.2.1 Personal Wellbeing Spectrum Questionnaire

A detailed description of the test can be found above and in Supplementary Appendix.

###### 2.3.1.2.2 Positivity Scale

The scale includes eight five-point Likert scale items that respondents rate based on how well they feel each statement describes them (1 = *Strongly disagree*; 5 = *Strongly agree*) (Caprara et al., 2012). The scale can assess the level of an individual’s positivity, which is conceptualized as capturing a general positive cognitive orientation toward oneself, one’s life, and one’s future (Caprara et al., 2017). Higher scores on POS reflect higher levels of positive attitude. POS was adapted for the Hungarian population by Oláh et al. (2025). In the present study Cronbach’s α: 0.902; McDonald’s ω: 0.915.

###### 2.3.1.2.3 Diener Flourishing Scale

This eight-item scale operationalizes an improved version of Diener’s concept of subjective wellbeing, in which, in addition to life satisfaction and the dominance of positive emotions, the necessity of competence, optimism, contributing to the wellbeing of others, life purpose, self-esteem, and positive relationships are also highlighted (Diener et al., 2010). For each item respondents had to rate on a seven-point Likert scale how much, in their view, they agreed with the statement corresponding to that item (1 = Strongly disagree; 7 = Strongly agree). A higher score on the scale indicates a higher level of the flourishing construct of subjective wellbeing. Diener’s test was adapted for the Hungarian population by Varga et al. (2025). In the present study Cronbach’s α: 0.932; McDonald’s ω: 0.934.

###### 2.3.1.2.4 Huppert Flourishing Scale

This scale is used to assess overall wellbeing and flourishing in individuals (Huppert and So, 2013). It consists of 10 items that evaluate different aspects of flourishing, such as positive relationships, feelings of competence, and sense of purpose. Items 1–7 are five-point graded, items 8 and 9 four-point graded, item 10 ten-point graded. Respondents had to rate for the statement of each item how much they agree with it (1 = Not at all; maximal value = Completely). For this reason, we first took the average of items 1 to 9 (*Huppert1_9*), and handled item 10 separately (*Huppert10*), which is an overall evaluation of happiness (Taking all things together, how happy would you say you are?). A higher score on the scale Huppert1_9 indicates a higher level of the flourishing construct of subjective wellbeing. A higher score on Huppert10 indicates a higher level of global happiness. For *Huppert1_9* in the present study we obtained Cronbach’s α: 0.872 and McDonald’s ω: 0.883. The one-dimensional factor structure of Huppert1_9 in Hungarian population was confirmed by Oláh (2021, Figure 4 and Table 4).

###### 2.3.1.2.5 Values in Action Inventory for Hungary

The original VIA questionnaire (Peterson and Seligman, 2004) contains 240 items, each representing one of 24 identified strengths, which are also used to assess six universal virtues. Furnham and Lester (2012) constructed a short, 24-item version of VIA, in which each of the 24 strengths was represented by only one item. For each item respondents had to rate on a six-point Likert scale how well, in their view, each statement was true of them. This latter version of VIA was adapted for the Hungarian population by Zábó et al. (2023). Based on explorative factor analyses, only four out of the six original virtues could be identified in the theoretical factor structure (*Wisdom and knowledge* with 5 items; *Humanity* with 5 items; *Temperance* with 3 items; *Spirituality and transcendence* with 7 items). The Hungarian version measures the 24 character strengths on a 6-point Likert scale (1 = *very much unlike me* to 6 = *very much like me*), with one positive item representing each strength (Zábó et al., 2023), just as in the version of Furnham and Lester (2012). Higher scores on each virtue scale indicate higher levels of the specific virtue. In Study I all virtue scales showed good reliability measured by Cronbach’s α (*Wisdom and knowledge*: 0.869; *Humanity*: 0.876; *Temperance*: 0.755; *Spirituality and transcendence*: 0.868) and McDonald’s ω (*Wisdom and knowledge*: 0.867; *Humanity*: 0.877; *Temperance*: 0.756; *Spirituality and transcendence*: 0.871). The VIA-H questionnaire data were collected only in the three subsamples of the 2019–2021 surveys (total number of cases: *n* = 1,713).

#### 2.3.2 Study II

##### 2.3.2.1 Participants and procedure

The survey was conducted among Hungarian-speaking individuals who were 18 years of age or older, yielding a sample of 1540 participants (390 males—25.4%, 1150 females—74.6%) with the mean age of 52.04 years (SD = 11.28 years, range: 18–86 years). 38.4% of the respondents had a high school degree or lower, 37.3% had a college/BA/BSc degree, and 22.5% had a university/MA/MSc degree (1.7% did not report the education level). When completing the questionnaire, all the questions had to be answered, and only then could they be submitted.

##### 2.3.2.2 Measures

Participants completed an 89-item online questionnaire. Ethical approval for the study was granted by the Research Ethics Committee of Eötvös Loránd University (permission number: 2019/21). Participation was voluntary and anonymous. Informed consent was obtained but no compensation was given.

The first 14 questions in the questionnaire (sociodemographic and mental health measurement variables), as well as the items of the PWBSQ and the DFS were the same as in Study I. In the present study we obtained for DFS Cronbach’s α: 0.938; McDonald’s ω: 0.938.

###### 2.3.2.2.1 Hungarian short form of the savoring beliefs inventory

The SBI-HU (Nagy et al., 2022) is a 10-item self-report questionnaire structured according to a three-factor model: anticipating (4 items), savoring the moment (3 items), and reminiscing (3 items). Participants were asked to report the extent of their agreement using a 7-point Likert scale (1 = strongly disagree; 7 = strongly agree). Higher scores indicated higher savoring capacity. In the present study we obtained for the total score: Cronbach’s α: 0.945; McDonald’s ω: 0.946, and for the three subscales (*Savoring via anticipation*, *Savoring the moment*, *Savoring via reminiscence*): Cronbach’s α: 0.896, 0.848, 0.850; McDonald’s ω: 0.897, 0.849, 0.851.

###### 2.3.2.2.2 Mental Health Test

The MHT (Zábó et al., 2022) is a 17-item self-report questionnaire, which operationalizes the theory of maintainable positive mental health. According to this theory the main pillars of positive mental health are wellbeing, efficient coping that enables an individual to maintain positive conditions and functioning, savoring capacity, resilience, and dynamic self-regulation. Participants were asked to report the extent of their agreement using a 6-point Likert scale (1 = strongly disagree; 6 = strongly agree). The five subscales of MHT are *wellbeing* (3 items), *Savoring* (3 items), *Creative and executive efficiency* (5 items); *Self-regulation* (3 items); *Resilience* (3 items). In Study II all scales showed good reliability measured by Cronbach’s α (*wellbeing*: 0.845; *Savoring*: 0.850; *Creative and executive efficiency*: 0.847; *Self-regulation*: 0.861; *Resilience*: 0.743) and McDonald’s ω (*Wellbeing*: 0.847; *Savoring*: 0.854; *Creative and executive efficiency*: 0.850; *Self-regulation*: 0.875; *Resilience*: 0.763).

###### 2.3.2.2.3 PERMA Questionnaire (PERMA-Profiler)

The PERMA model was developed by Seligman (2018), building on his earlier concept of authentic happiness. The components of the five-pillar model reinforce one another in creating and maintaining a state of wellbeing. The 23-item PERMA-Profiler (Butler and Kern, 2016) measures Seligman’s model using a 10-point Likert scale (0 = *never/not at all/terrible*; 10 = *always/completely/excellent*). In the case of 15 items, the five basic pillars (P = *Positive emotions*, E = *Engagement*, R = *Relationships*, M = *Meaning*, A = *Accomplishment*) are measured using three questions each; six items, comprising three questions each, are used to assess *negative emotions* (N) and *health* (H). Among the 23 items one item is used to measure *happiness* and one item is used to measure *loneliness*. PERMA was adapted for the Hungarian population by Varga et al. (2022) on this sample of Study II. In Study II all but one scale showed good reliability measured by Cronbach’s α (*Positive emotions*: 0.883; *Engagement*: 0.558; *Relationships*: 0.791; *Meaning*: 0.762; *Accomplishment*: 0.738; *Health*: 0.879; *Negative emotions*: 0.771) and McDonald’s ω (*Positive emotions*: 0.883; *Engagement*: 0.592; *Relationships*: 0.793; *Meaning*: 0.782; *Accomplishment*: 0.779; *Health*: 0.880; *Negative emotions*: 0.780).

#### 2.3.3 Study III

##### 2.3.3.1 Participants and procedure

The survey was conducted among Hungarian-speaking individuals who were 18 years of age or older, yielding a sample of 635 participants (64 males—10.1%, 571 females) with the mean age of 51.82 years (SD = 11.12 years, range: 20–80 years). 40.2% of the respondents had a high school degree or lower, 34.0% had a college/BA/BSc degree, and 25.8% had a university/MA/MSc degree. When completing the questionnaire, all the questions had to be answered, and only then could they be submitted.

##### 2.3.3.2 Measures

Three of the questions in the questionnaire referred to sociodemographic data (gender, age, and education level. Participants completed a 61-item online questionnaire. Ethical approval for the study was granted by the Research Ethics Committee of Eötvös Loránd University (permission number: 2020/21). Participation was voluntary and anonymous. Informed consent was obtained but no compensation was given. The items of the PWBSQ were the same as in Study I.

###### 2.3.3.2.1 Mental Health Test

MHT was the same as in Study II. In Study III all scales showed good reliability measured by Cronbach’s α (Wellbeing: 0.831; Savoring: 0.768; Creative and Executive Efficiency: 0.803; Self-regulation: 0.733; Resilience: 0.774) and McDonald’s ω (Wellbeing: 0.833; Savoring: 0.770; Creative and Executive Efficiency: 0.813; Self-regulation: 0.736; Resilience: 0.796).

###### 2.3.3.2.2 Values in Action Inventory for Hungary

VIA-H was the same as in Study I. In Study III all scales showed good reliability measured by Cronbach’s α (Wisdom and knowledge: 0.872; Humanity: 0.874; Temperance: 0.768; Spirituality and transcendence: 0.852) and McDonald’s ω (Wisdom and knowledge: 0.871; Humanity: 0.876; Temperance: 0.770; Spirituality and transcendence: 0.854).

#### 2.3.4 Study IV

##### 2.3.4.1 Participants and procedure

The survey was conducted among Hungarian-speaking individuals living in the Transylvania region of Romania, who were 18 years of age or older, yielding a sample of 1044 participants (288 males—27.6%, 756 females—72.4%) with the mean age of 37.87 years (SD = 13.70 years, range: 18–89 years). 39.2% of the respondents had a high school degree or lower, 0.8% had a college/BA/BSc degree, and 60.0% had a university/MA/MSc degree. When completing the questionnaire, all the questions had to be answered, and only then could they be submitted.

##### 2.3.4.2 Measures

Participants completed a 67-item online questionnaire. Ethical approval for the study was granted by the Research Ethics Committee of Eötvös Loránd University (permission number: 2020/21). Data collection was conducted by Tímea Krizbai and István Zsigmond from Sapientia Hungarian University of Transylvania. Participation was voluntary and anonymous. Informed consent was obtained but no compensation was given.

The first 14 questions in the questionnaire (sociodemographic and mental health measurement variables), as well as the items of the PWBSQ, POS, and DFS were the same as in Study I. In the present study we obtained for POS Cronbach’s α: 0.881; McDonald’s ω: 0.897, and for DFS Cronbach’s α: 0.909; McDonald’s ω: 0.910.

###### 2.3.4.2.1 Huppert Flourishing Scale

HFS was the same as in Study I. Because of the largely differing scales of the items, we again formed on the one hand the average of items 1 to 9 (*Huppert1_9*), and treated separately item 10 (*Huppert10*), an overall evaluation of happiness. In Study IV we obtained for *Huppert1_9* Cronbach’s α: 0.832 and McDonald’s ω: 0.844.

###### 2.3.4.2.2 Hungarian short form of the savoring beliefs inventory

The SBI-HU was the same as in Study II. In Study IV we obtained for the total score: Cronbach’s α: 0.912; McDonald’s ω: 0.913, and for the three subscales (*Savoring via anticipation*, *Savoring the moment*, *Savoring via reminiscence*): Cronbach’s α: 0.835, 0.779, 0.821; McDonald’s ω: 0.836, 0.78.822.

### 2.4 Statistical analyses

The structural validity of PWBSQ was examined by a series of confirmatory factor analysis (CFA). The best final factor model was identified by the total sample from Study I, and this model was tested on samples from Studies II to IV. In CFA we chose a robust method for model fitting (maximum likelihood mean variance, MLMV), which, in the case of CFA, provides a good alternative to the traditional ML method requiring multivariate normality (Gao et al., 2020). For this purpose, the CFA module of statistical software ROP-R (Vargha and Bánsági, 2022) was used. It is based on the *cfa* function of the *lavaan* package of R (Rosseel, 2012) with the mimic = “Mplus” parametrization, which provides an output comparable to that of Mplus software (Muthén and Muthén, 1998–2011).

To assess the global fit of the CFA models, the following fit indices were used, with the cut-off values in parentheses: Comparative fit index—CFI (> 0.90), Tucker–Lewis index—TLI (> 0.90), root mean squared error of approximation—RMSEA (< 0.08), standardized root mean squared error—SRMR (< 0.08) (Hu and Bentler, 1999; van de Schoot et al., 2012). A significant χ^2^ test indicates an inadequate model fit. However, the significance of this test is highly sensitive to sample size, easily rendering the χ^2^ test statistic significant (Alavi et al., 2020; Byrne, 2016).

Along with CFA we performed measurement invariance analyses for five categorical sociodemographic variables, using the multigroup CFA framework (He and van de Vijver, 2012; Milfont and Fischer, 2010; van de Schoot et al., 2012). To test invariance, configural, metric and scalar models were compared (Bowen and Guo, 2012). The configural invariance model did not contain any constraints on factor loadings and intercepts. In the metric model the factor loadings were set to be equal across groups but on the intercepts there were no constraints. For testing scalar invariance, factor loadings and intercepts were assumed to be equal across groups. Measurement invariance was performed using Mplus software (Muthén and Muthén, 1998–2011).

In measurement invariance, the standard procedure is to use the χ^2^ difference test with a significant Δχ^2^ indicating non-invariance, but again, the significance is highly sensitive to sample size (Gana and Broc, 2019). For this reason, our decision about invariance was primarily based on ΔCFI (with the threshold being the value greater than 0.01; Cheung and Rensvold, 2002) and ΔRMSEA (greater than 0.015; Putnick and Bornstein, 2016; Beribisky and Hancock, 2023; Chen, 2007), comparing the model fit indices with and without equality constraints in the configural vs. metric and metric vs. scalar relations.

In assessing the effects of a sociodemographic group, the type of invariance should be set to scalar in Mplus. The significances of the effect sizes are indicated by the significances of the group means, which are always compared to the reference group (the default is the first group), and Cohen’s *d* values can be computed from the mean and variance estimates. In assessing these effects, we also performed ANOVAs. In ANOVAs Cohen’s *d* and eta-squared (η^2^) indicators were calculated as effect size measures. Following Cohen (1988) 1992, the effect size of *d* is considered small, medium (moderate) or large if *d* is around 0.2, 0.5, 0.8 or higher. Cohen (1988) also proposed an interpretation of the η^2^ indicator with thresholds of 0.01 (small), 0.06 (medium), 0.14 (large).

The internal consistency of the scales was assessed using Cronbach’s α and McDonald’s ω (McDonald, 1999; Dunn et al., 2014). The construct and content validity were inspected using Pearson and Spearman correlations. Regarding the classification of the strength of the Pearson’s correlations, we followed Cohen’s convention (Cohen, 1988, p. 79–80). According to this, a correlation is said to be weak if the absolute value of *r* is less than 0.3 but reaches 0.1, and to be medium, strong, or very strong if the absolute value of *r* reaches 0.3, 0.5, or 0.7, respectively. Since the Spearman correlation is a Pearson correlation calculated on ranks (Astivia and Zumbo, 2017), it was evaluated using the same thresholds. Statistical analyses were performed by Mplus (Muthén and Muthén, 1998–2011), ROPstat (Vargha et al., 2015), ROP-R (Vargha and Bánsági, 2022), and SPSS (IBM Corp, 2020).

## 3 Results

### 3.1 Structural validity of PWBSQ in Study I

First, we performed a first order four-factor CFA in the total sample, which tested a four-dimensional structure of PWBSQ. The chi-square goodness of fit test was highly significant (χ^2^ = 6855.23, *df* = 113, *p* < 0.001), but this is usual in case of large samples like ours. A sequential fit diagnostic evaluation indicated that the points of ill fit pertained partly to the error covariances of Items 1 and 2 (see Supplementary Appendix). Incorporating this covariance into the factor model, the chi-square goodness of fit test was still highly significant, but the additional CFA fit measures were acceptable (see first row of Table 3).

The factors in the first-order model are all highly correlated (see Table 4). Therefore, it seems reasonable to assume that they are components of one or two second order factors. Since the estimated correlation between latent factors *Emot* and *Psych* (*r* = 0.935) and between latent factors *Soc* and *Spirit* (*r* = 0.938) stand out among these correlations, we tested a second order CFA model with two second order latent factors determining the first-order factors *Emot* and *Psych*, and *Soc* and *Spirit* separately.

**TABLE 4:** Pairwise correlation estimates of the latent factors of the first order model.

Latent factor	*Psych*	*Soc*	*Spirit*
*Emot*	0.935	0.761	0.733
*Psych*		0.830	0.803
*Soc*			0.938

Since both *Emot* and *Psych* are considered as internal, self-focused components of wellbeing, and *Soc* and *Spirit* are considered as external-focused components of wellbeing, the second order factor determining the former couple was called the *internal wellbeing* factor (*WBint*) and the second order factor determining the latter couple was called the *external wellbeing* factor (*WBext*). The results of this CFA indicated that the fitting of this second order model was just acceptable as the first-order model (see second row of Table 3).

Nevertheless, this second order model had a serious flaw, as the estimated variance of latent factor *Psych* was negative (−0.021). Therefore, we tried to improve the model by omitting one item from this first-order factor. The correction was the most successful for item 15 (i15psy) of the questionnaire. Since in this case the absolute fit index RMSEA does not exceed 0.06, and the relative fit indices CFI and TLI are both above 0.95, this factor model can be considered good (see third row of Table 3). It is worth noting that the omission of item 15 also improves the first order model, which is practically just as good as the second order model (see the last but one row of Table 3). However, performing a first order one-factor CFA with these 16 items, not sorting them into the four scales, the model fit becomes unacceptable (see last row of Table 3).

**FIGURE 1:**
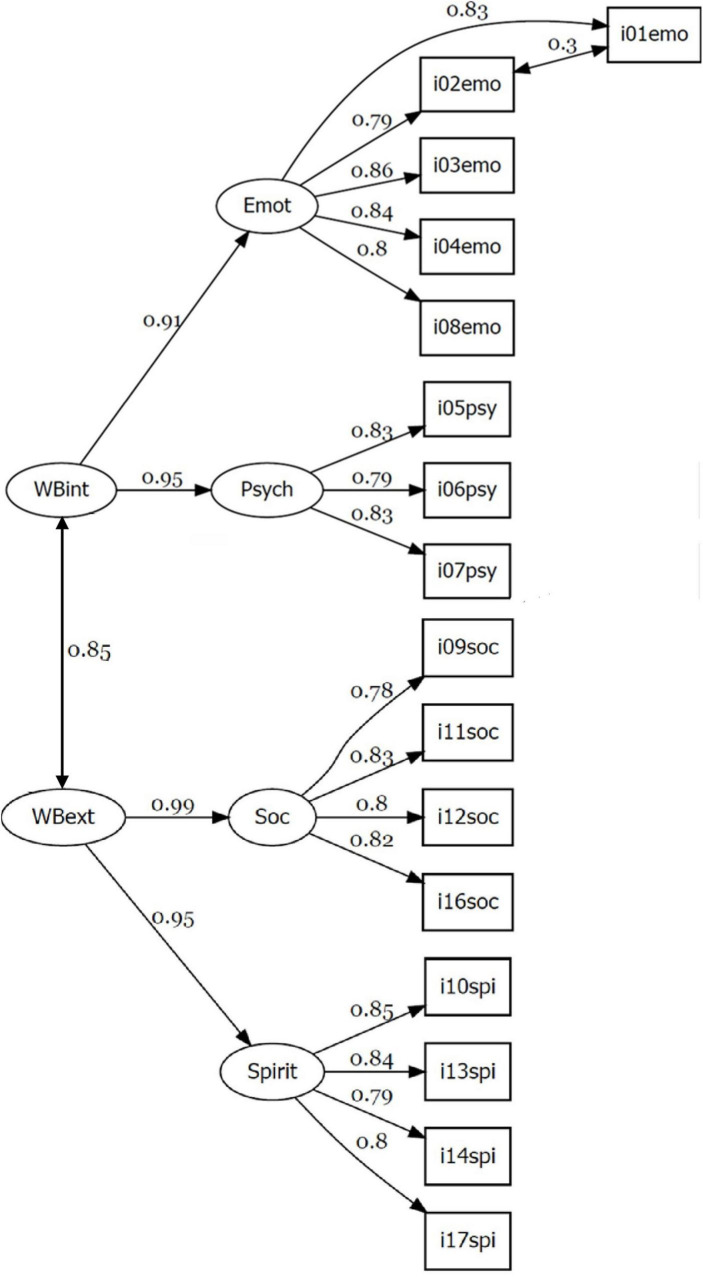
The path diagram of the final second order CFA model with the standardized regression estimates in the total sample of Study I (*n* = 11,686).

The standardized regression estimates (rounded to two decimals) of the final best model can be seen in Figure 1. The factor loadings are all acceptable (above 0.75). Although the correlation estimation (0.85) between the two second order factors is very strong, the shared variance (*r*^2^ = 0.72) indicates that their psychological content can be different. The allowed correlation between items i01emo and i02emo (0.30) is, however, small.

### 3.2 Confirmation of the second order factor structure identified in Study I

We confirmed the improved second order factor structure of the 16-item PWBSQ identified in Study I (see Figure 1 and the third row of Table 3) by means of samples of Studies II to IV. The results summarized in Table 5 show that the fit of the factor structure identified in Study I was found to be acceptable in all other samples.

**TABLE 5:** Adequacy measures of the final second order CFA model in Studies I to IV.

Study	Sample size	χ^2^	RMSEA	CI_90_(RMSEA)	pClose	CFI	TLI	SRMR
I	11,686	4282.39	0.060	(0.059;0.062)	< 0.001	0.960	0.951	0.032
II	1,540	756.49	0.066	(0.062;0.071)	< 0.001	0.947	0.935	0.043
III	635	303.79	0.058	(0.050;0.065)	0.044	0.955	0.945	0.039
IV	1,044	388.23	0.055	(0.049;0.060)	0.086	0.952	0.941	0.040

In the table all χ^2^ values are significant at *p* < 0.001 level (*df* = 98).

### 3.3 Internal consistency of the PWBSQ scales

Based on the confirmed second order factor structure detailed in section 3.2, we are justified to define four scales corresponding to the four first order factors (*Emot*, *Psych*, *Soc*, and *Spirit*), and two scales corresponding to the two second order factors (*WBint* and *WBext*). A *Personal wellbeing* total score scale, based on the high correlation between the two second order factors (see Figure 1) may also seem to be reasonable, but the one-dimensional structure of the PWBSQ was not supported by the CFA (see last row of Table 3). The internal consistency of these six scales of PWBSQ was examined by means of the *Reliability measures* option of the Principal Component Analysis module of ROP-R, which is based on the MBESS package of R (Kelley, 2007). As shown in Tables 6, 7, the internal consistency is excellent in each study for all scales. These results, together with the CFA results, confirm the reliability of the four first order and the two second order scales of PWBSQ in the Hungarian population.

**TABLE 6:** Cronbach’s α values measuring the internal consistency of the PWBSQ scales.

PWBSQ scale	Study I	Study II	Study III	Study IV
*Emotional wellbeing* (*Emot*)	0.916	0.923	0.910	0.871
*Psychological wellbeing* (*Psych*)	0.856	0.863	0.847	0.792
*Social wellbeing* (*Soc*)	0.884	0.892	0.878	0.864
*Spiritual wellbeing* (*Spirit*)	0.892	0.906	0.900	0.856
*Internal wellbeing* (*WBint*)	0.932	0.938	0.927	0.893
*External wellbeing* (*WBext*)	0.934	0.938	0.936	0.915

**TABLE 7:** McDonald’s ω values measuring the internal consistency of the PWBSQ scales.

PWBSQ Scale	Study I	Study II	Study III	Study IV
*Emotional wellbeing* (*Emot*)	0.916	0.924	0.911	0.872
*Psychological wellbeing* (*Psych*)	0.860	0.864	0.847	0.795
*Social wellbeing* (*Soc*)	0.885	0.894	0.881	0.867
*Spiritual wellbeing* (*Spirit*)	0.893	0.906	0.900	0.856
*Internal wellbeing* (*WBint*)	0.932	0.939	0.928	0.894
*External wellbeing* (*WBext*)	0.934	0.939	0.937	0.916

### 3.4 Measurement invariance of PWBSQ

Measurement invariance cannot be performed with higher order models in Mplus. For this reason, we tested measurement invariance with the final first order model, which is just as good as the final second order model (see 3rd and 4th row of Table 3). Measurement invariance of PWBSQ was assessed in the largest Study I sample for categorical sociodemographic variables of gender, age level, education, occupation, and marital status (see Table 2). Results concerning this analysis are summarized in Table 8.

**TABLE 8:** Measurement invariance of PWBSQ for five sociodemographic variables.

Variable	CFI	TLI	RMSEA	SRMR	MC	ΔRMSEA	ΔCFI
**Gender**							
1. Configural	0.958	0.948	0.061	0.033			
2. Metric	0.958	0.951	0.060	0.034	2 vs. 1	0.001	0
3. Scalar	0.957	0.952	0.059	0.027	3 vs. 2	0.001	0.001
**Age level**							
1. Configural	0.958	0.948	0.062	0.034			
2. Metric	0.956	0.951	0.060	0.036	2 vs. 1	0.002	0.002
3. Scalar	0.947	0.945	0.063	0.040	3 vs. 2	0.003	0.009
**Education**							
1. Configural	0.958	0.948	0.058	0.034			
2. Metric	0.957	0.951	0.056	0.037	2 vs. 1	0.002	0.001
3. Scalar	0.954	0.952	0.055	0.038	3 vs. 2	0.001	0.003
**Occupation**							
1. Configural	0.956	0.945	0.059	0.036			
2. Metric	0.954	0.948	0.058	0.041	2 vs. 1	0.001	0.002
3. Scalar	0.938	0.935	0.064	0.047	3 vs. 2	0.006	0.016
**Marital status**							
1. Configural	0.958	0.948	0.058	0.034			
2. Metric	0.957	0.951	0.056	0.036	2 vs. 1	0.002	0.001
3. Scalar	0.952	0.951	0.057	0.038	3 vs. 2	0.001	0.005

The following fit indices are reported: Comparative fit index (CFI); Tucker-Lewis Index (TLI); Root Mean Square Error of Approximation (RMSEA), Standardized Root Mean Square Residual (SRMR), MC, model comparison.

A CFA model can be accepted if the relative fit indices CFI and TLI are greater than 0.90 (CFI_*min*_ = 0.938; TLI_*min*_ = 0.935), and the absolute fit indices RMSEA and SRMR are all less than 0.08 (RMSEA_*max*_ = 0.064; SRMR_*max*_ = 0.047). In this respect all measurement models of Table 8 can be accepted. Models never get worse substantially from a lower level to a higher level one, because all ΔRMSEA values are less than 0.15 (ΔRMSEA_*max*_ = 0.06), and all but one ΔCFI value is less than 0.10. A weak violation of invariance at the scalar level was only obtained in the metric vs. scalar comparison for occupation indicated by a slightly elevated ΔCFI value (0.016).

### 3.5 Basic statistics of the PWBSQ scales

In sections 3.1 and 3.2, the stable second-order factor structure of PWBSQ with four first-order factors in different samples was demonstrated. Based on this, it is justified to use first-order scales corresponding to the first-order factors (*Emot*, *Psych*, *Soc*, *Spirit*) according to the scale membership (omitting i15psy) indicated in Supplementary Appendix by averaging the items. In addition, it is also legitimate to use scales defined by the items corresponding to the two second-order factors (*WBint* as the average of the items of the *Emot* and *Psych* scales, and *WBext* as the average of the items of the *Soc* and *Spirit* scales). The use of these scales is also confirmed by the good reliability data of these scales (Tables 6, 7). The basic statistics of these scales in the different samples of the four studies are summarized in Table 9.

**TABLE 9:** Basic statistics of the PWBSQ scales.

Scale	*Emot*	*Psych*	*Soc*	*Spirit*	*WBint*	*WBext*
**Study I (n = 11,686)**						
Median	4.00	4.33	4.00	4.00	4.25	4.00
Mean	4.01	4.33	3.81	3.97	4.13	3.89
SD	1.14	1.09	1.23	1.26	1.06	1.19
Skewness	−0.40	−0.65	−0.29	−0.36	−0.52	−0.33
Kurtosis	−0.42	0.21	−0.65	−0.60	−0.16	−0.59
**Study II (n = 1,540)**						
Median	4.20	4.67	4.00	4.50	4.25	4.38
Mean	4.15	4.43	4.01	4.40	4.26	4.20
SD	1.03	1.00	1.18	1.17	0.97	1.12
Skewness	−0.35	−0.61	−0.32	−0.61	−0.44	−0.45
Kurtosis	−0.17	0.28	−0.51	−0.18	0.00	−0.34
**Study III (n = 635)**						
Median	4.20	4.33	4.00	4.25	4.25	4.13
Mean	4.04	4.32	3.86	4.22	4.15	4.04
SD	0.99	0.96	1.14	1.17	0.92	1.11
Skewness	−0.44	−0.59	−0.32	−0.54	−0.58	−0.43
Kurtosis	−0.06	0.54	−0.39	−0.19	0.25	−0.25
**Study IV (n = 1,044)**						
Median	4.00	4.33	4.00	4.00	4.00	4.00
Mean	4.04	4.24	3.88	3.97	4.11	3.92
SD	0.93	0.91	1.06	1.08	0.85	1.02
Skewness	−0.05	−0.16	−0.28	−0.28	−0.06	−0.23
Kurtosis	−0.17	0.06	−0.27	−0.24	−0.03	−0.21

Based on Table 9, it is worth noting that although normality is violated, the extent of the violation cannot be considered severe due to the low absolute values (below 1) of skewness and kurtosis (Curran et al., 1996). The intercorrelations of the PWBSQ scales in Study I—all very strong according to Cohen’s convention, and significant at *p* < 0.001 level—are summarized in Table 10. Similar correlations were also obtained in Studies II, III, and IV.

**TABLE 10:** Intercorrelations of the PWBSQ scales in Study I.

Scale	*Emot*	*Psych*	*Soc*	*Spirit*	*WBint*
*Psych*	0.770	0.693	0.822	0.704	0.750
*Soc*	0.686				
*Spirit*	0.663	0.669			
*WBint*	0.969	0.904	0.728		
*WBext*	0.707	0.713	0.953	0.956	

### 3.6 Internal validity of PWBSQ domains

Empirical results from four studies provide robust support for the theoretical framework of the PWBST and its implementation through the PWBSQ. The initial four-factor structure—emotional, psychological, social and spiritual wellbeing—was confirmed through first-order CFA in Study I, yielding acceptable fit indices (see Table 3, row 1). However, the exceptionally high correlations among these dimensions (Table 4) suggested a deeper latent organization.

This led to the development of a second-order CFA model which grouped the emotional and psychological dimensions under an internal wellbeing factor (*WBint*) and the social and spiritual dimensions under an external wellbeing factor (*WBext*). Excluding item i15psy, the refined second-order model demonstrated good fit (Table 3, row 3) and was subsequently validated across three independent samples (Studies II–IV) with consistently strong fit indices (Table 5). These findings support the theoretical proposition that personal wellbeing is best conceptualized as a dynamic interplay between internal and external domains of human functioning.

The internal consistency of the PWBSQ scales further reinforces the instrument’s structural validity. Cronbach’s α and McDonald’s ω values were uniformly high in all samples (Tables 6, 7) across all six scales—four first-order and two second-order—indicating reliable measurement of the underlying constructs. Notably, the second-order scales (*WBint* and *WBext*) exhibited internal consistency values exceeding 0.93 in the largest sample (Study I), highlighting their psychometric robustness.

Measurement invariance analyses confirmed that the PWBSQ performs consistently across key sociodemographic groups, including gender, age, education, occupation, and marital status (see Table 8). Configural, metric, and scalar models all demonstrated an acceptable fit, with only minor changes in fit indices observed across levels. Only a marginal violation of scalar invariance was observed for occupation (ΔCFI = 0.016), which does not substantially compromise the interpretability of group comparisons.

Descriptive statistics for the PWBSQ scales (Table 9) revealed generally elevated wellbeing scores across the samples, with means consistently above the midpoint of the scales and acceptable variability. Although normality assumptions were technically violated, the low absolute values of skewness and kurtosis suggest that these deviations are not severe (Curran et al., 1996). The intercorrelations among the scales (Table 10) were uniformly strong. Notably, there were particularly high associations between *WBint* and its components (*Emot*: *r* = 0.969; *Psych*: *r* = 0.904), as well as between *WBext* and its components (*Soc*: *r* = 0.953; *Spirit*: *r* = 0.956). This confirms the coherence of the second-order structure.

Based on the validated second-order factor structure of the PWBSQ (see sections 3.1–3.2 and Table 3), two composite scales were derived that reflect the two overarching domains of personal wellbeing: *WBint* and *WBext*. *WBint* captures self-referential aspects of wellbeing, integrating emotional regulation, psychological resilience, and personal growth. *WBext* encompasses relational and transcendent dimensions, including social connectedness, community integration, and spiritual meaning.

These composite scales were operationalized by averaging the items corresponding to their respective first-order factors. *WBint* includes items from the Emotional and Psychological wellbeing scales, while *WBext* includes items from the Social and Spiritual wellbeing scales. The formation of these scales is theoretically and empirically justified—based on the conceptual coherence of the second-order model—as evidenced by their good reliability indices (see Tables 6, 7), and their strong correlations with their respective component scales (Table 10).

Taken together, these findings provide convergent empirical support for the PWBST and its multidimensional operationalization through PWBSQ. The emergence of a dual-spectrum structure (internal and external wellbeing) not only enhances the psychometric clarity of the instrument but also offers a novel theoretical lens for understanding personal wellbeing. This integrative framework holds promise for future research and intervention design, particularly in contexts that require nuanced assessment of individual flourishing across diverse domains of human experience.

### 3.7 External and content validity of the PWBSQ scales

The content validity of the PWBSQ scales was analyzed with Pearson’s correlations using the six scales of PWBSQ and different variables of positivity and physical or mental health of the different studies. Table 11 shows the correlation results for the variables of Study I. To see the difference between the meaning of the *WBint* and *WBext* scales, we also calculated the partial correlations that filter out the linear effect of each other in the correlations with these variables (see the last two columns in Table 11).

**TABLE 11:** Pearson’s correlations of the PWBSQ scales with other positivity and mental health measurement indices from Study I (*n* = 11,686; all correlations and partial correlations above 0.030 are significant at *p* < 0.001).

Variable	*Emot*	*Psych*	*Soc*	*Spirit*	*WBint*	*WBext*	*WBint^a^*	*WBext^b^*
GMst	0.749	0.596	0.544	0.521	0.733	0.558	0.574	0.017
ActH	0.699	0.533	0.519	0.511	0.676	0.539	0.487	0.067
Pexp%	0.664	0.500	0.473	0.449	0.639	0.483	0.478	0.007
PhysC	0.491	0.424	0.388	0.355	0.493	0.389	0.331	0.032
Health	0.533	0.452	0.418	0.386	0.533	0.421	0.362	0.038
PhysStr	0.512	0.482	0.434	0.404	0.530	0.439	0.338	0.074
POS	0.790	0.693	0.638	0.618	0.798	0.658	0.612	0.148
DFS	0.737	0.701	0.674	0.642	0.765	0.689	0.519	0.270
H1_9	0.766	0.705	0.646	0.634	0.786	0.671	0.578	0.198
H10	0.785	0.607	0.577	0.554	0.762	0.592	0.596	0.048

GMst, *General mental state*; ActH, *Actual happiness*; Pexp%, *Positive experience%*; PhysC, *Physical condition*; Health, *General health condition*; PhysStr, *Physical strength*; POS, *Positivity Scale*; DFS, *Diener Flourishing Scale*; H1_9, *Huppert1_9*; H10, *Huppert10* (*Global happiness*).

*^a^*Column contains correlations with *WBint* after partialling out the linear effect of *WBext.*

*^b^*Column contains correlations with *WBext* after partialling out the linear effect of *WBint.*

The following conclusions can be drawn from Table 11.

1. Correlations with the four basic scales of the PWBSQ (*Emot*, *Psych*, *Soc*, and *Spirit*) are always monotonically decreasing for each variable.
2. For this reason, the social and spiritual components of wellbeing are less prominent in the content of the flourishing and positivity scales, and the simple items of actual and global happiness and physical and mental states, than the emotional (biological) and psychological components.
3. Consequently, the correlation with *WBint* is always considerably higher than the correlation with *WBext* for the same variable.
4. Among the row variables, general mental state, POS, the Diener Flourishing Scale and both measures of the Huppert Flourishing Scale (Huppert1_9 and Huppert10) are in a very strong relationship with *Emot* and *WBint* (*r* > 0.73), but the other variables correlate also strongly (*r* > 0.50) with *Emot* and *WBint*, or close to this level (for physical condition *r* > 0.49).
5. If we partial out the linear effect of *WBext* from the correlations with *WBint*, the correlations remain strong (*r* > 0.50).
6. However, if the linear effect of *WBint* is removed from the correlations with *WBext*, the correlations often fall to unexplainable levels (*r* < 0.10), as in the case of all physical and mental state measures, and actual and global happiness. Only for test variables POS, DFS and Huppert1_9 is true that the partial correlation with *WBext* after removing the linear effect of *WBint* does not fall under 0.1. The highest among them is with DFS (*r*_*partial*_ = 0.27. close to the medium level).

Table 12 summarizes the correlations of the PWBSQ scales with VIA-H virtue scales from Study I. It is interesting that the virtue scales do not correlate stronger with *WBint* than with *WBext*. This is quite natural, because the main types of virtues rely just as much on social and spiritual grounds as on biological and psychological ones.

**TABLE 12:** Pearson’s correlations of the PWBSQ scales with VIA-H virtue scales from Study I (*n* = 1.713).

Variable	*Emot*	*Psych*	*Soc*	*Spirit*	*WBint*	*WBext*	*WBint^a^*	*WBext^b^*
Wisdom	0.440**	0.541**	0.445**	0.458**	0.505**	0.473**	0.259**	0.166**
Human	0.462**	0.480**	0.482**	0.507**	0.496**	0.518**	0.191**	0.256**
Temper	0.350**	0.349**	0.328**	0.360**	0.370**	0.361**	0.162**	0.136**
STransc	0.570**	0.552**	0.558**	0.597**	0.597**	0.605**	0.273**	0.299**

Wisdom, *Wisdom and knowledge*; Human, *Humanity*; Temper, *Temperance*; STransc, *Spirituality and transcendence*;

**p < 0.01.

*^a^*Column contains correlations with *WBint* after partialling out the linear effect of *WBext.*

*^b^*Column contains correlations with *WBext* after partialling out the linear effect of *WBint.*

Table 13 summarizes the correlations of the PWBSQ scales with other positivity and mental health measurement indices from Study II. The correlations with physical and mental health measures and DFS in the first nine lines show the same patterns as in Study I (see Table 11), showing the dominance of *WBint* compared to *WBext*. However, the Savoring scales in the next four lines resemble more to the virtue scales of Study I (see Table 12), which are almost as strongly related to the social and spiritual components of wellbeing as to the biological and psychological ones. The next five MHT scales show a varied correlation pattern. The pattern of wellbeing subscale (MWB) is very similar to that of *General mental state* in Study I and Study II and POS and Huppert10 in Study I. The Savoring subscale (MSav) behaves similarly to Savoring scales above, and the *Creative and executive efficiency* subscale (MCeff) shows a balanced relationship with the two main second order pillars of wellbeing. The *Self-regulation* and *Resilience* subscales, however, relate again more strongly to *WBint* than to *WBext*, with a similar correlation pattern as physical state and health measures above. Finally, the PERMA scales and indices relate again more strongly to *WBint* than to *WBext*, but at different correlation levels. One exception is the *Engagement* scale (E), where the correlations with the different wellbeing components, and the two partial correlations are of the same level. For the other PERMA scales and indices, after removing the linear effect of *WBint*, the correlation with *WBext* drops below 0.11 in absolute value.

**TABLE 13:** Pearson’s correlations of the PWBSQ scales with other positivity and mental health measurement indices from Study II (*n* = 1,540).

Variable	*Emot*	*Psych*	*Soc*	*Spirit*	*WBint*	*WBext*	*WBint^a^*	*WBext^b^*
GMst	0.755**	0.630**	0.503**	0.487**	0.744**	0.519**	0.626**	−0.058*
ActH	0.648**	0.519**	0.427**	0.427**	0.631**	0.447**	0.499**	−0.028
Pexp%	0.489**	0.379**	0.327**	0.324**	0.471**	0.341**	0.346**	−0.007
PhysC	0.513**	0.438**	0.335**	0.322**	0.510**	0.344**	0.403**	−0.050*
Health	0.547**	0.473**	0.360**	0.343**	0.546**	0.368**	0.437**	−0.056*
PhysStr	0.518**	0.494**	0.371**	0.354**	0.535**	0.380**	0.407**	−0.020
DFS	0.778**	0.762**	0.693**	0.678**	0.811**	0.719**	0.601**	0.313**
Savfut	0.605**	0.586**	0.532**	0.609**	0.628**	0.597**	0.349**	0.259**
Savpres	0.650**	0.641**	0.558**	0.621**	0.679**	0.617**	0.424**	0.240**
Savpast	0.527**	0.495**	0.463**	0.518**	0.541**	0.514**	0.282**	0.206**
Savor	0.634**	0.612**	0.553**	0.622**	0.658**	0.615**	0.385**	0.261**
MWB	0.798**	0.665**	0.564**	0.556**	0.787**	0.587**	0.648**	0.024
MSav	0.546**	0.509**	0.456**	0.513**	0.559**	0.508**	0.319**	0.174**
MCeff	0.581**	0.652**	0.556**	0.557**	0.638**	0.583**	0.380**	0.222**
MSreg	0.433**	0.387**	0.284**	0.298**	0.437**	0.305**	0.330**	−0.025
Mresil	0.575**	0.508**	0.392**	0.386**	0.578**	0.408**	0.450**	−0.029
P	0.817**	0.696**	0.579**	0.559**	0.812**	0.596**	0.687**	0.003
E	0.419**	0.441**	0.387**	0.420**	0.449**	0.423**	0.226**	0.154**
R	0.643**	0.529**	0.534**	0.442**	0.631**	0.512**	0.439**	0.093**
M	0.736**	0.730**	0.593**	0.573**	0.771**	0.611**	0.600**	0.107**
A	0.644**	0.662**	0.507**	0.449**	0.683**	0.501**	0.537**	0.001
N	−0.636**	−0.521**	−0.434**	−0.420**	−0.624**	−0.448**	−0.486**	0.017
H	0.538**	0.489**	0.350**	0.342**	0.546**	0.363**	0.442**	−0.066**
PHappy	0.780**	0.640**	0.546**	0.517**	0.765**	0.557**	0.632**	−0.008
PLonely	−0.483**	−0.381**	−0.316**	−0.277**	−0.468**	−0.311**	−0.371**	0.053*

GMst, *General mental state*; ActH, *Actual happiness*; Pexp%, *Positive experience%*; PhysC, *Physical condition*; Health, *General health condition*; PhysStr, *Physical strength*; POS, *Positivity Scale*; DFS, *Diener Flourishing Scale*; Savfut, *Savoring via anticipation*; Savpres, *Savoring the moment*; Savpast, *Savoring via reminiscence*; Savor, *Savoring Total*; MWB, *MHT WellBeing*; MSav, *MHT Savoring*; MCeff, *MHT Creative and executive efficiency*; MSreg, *MHT Self-regulation*; Mresil, *MHT* Resilience; P, *Positive emotions*; E, *Engagement*; R, *Relationships*; M, *Meaning*; A, *Accomplishment*; N, *Negative emotions*; H, *Health*; PHappy, *Happiness*; PLonely, *Loneliness*;

*p < 0.05,

**p < 0.01.

*^a^*Column contains correlations with *WBint* after partialling out the linear effect of *WBext.*

*^b^*Column contains correlations with *WBext* after partialling out the linear effect of *WBint.*

Table 14 summarizes the correlations of the PWBSQ scales with mental health and virtue scales from Study III. The MHT subscales show the same correlation patterns as in Study II, and the VIA-H virtue scales show the same correlation patterns as in Study I.

**TABLE 14:** Pearson’s correlations of the PWBSQ scales with mental health and virtue scales from Study III (*n* = 635).

Variable	*Emot*	*Psych*	*Soc*	*Spirit*	*WBint*	*WBext*	*WBint^a^*	*WBext^b^*
MWB	0.784**	0.595**	0.561**	0.514**	0.758**	0.560**	0.616**	0.014
MSav	0.425**	0.402**	0.393**	0.415**	0.442**	0.421**	0.216**	0.160**
MCeff	0.446**	0.561**	0.519**	0.487**	0.518**	0.524**	0.232**	0.249**
MSreg	0.303**	0.249**	0.219**	0.201**	0.301**	0.219**	0.211**	−0.001
Mresil	0.504**	0.434**	0.410**	0.340**	0.507**	0.391**	0.353**	0.034
Wisdom	0.388**	0.520**	0.411**	0.434**	0.463**	0.441**	0.229**	0.170**
Human	0.343**	0.384**	0.372**	0.382**	0.380**	0.393**	0.148**	0.182**
Temper	0.310**	0.331**	0.275**	0.269**	0.337**	0.284**	0.199**	0.058
STransc	0.524**	0.490**	0.477**	0.529**	0.542**	0.525**	0.273**	0.224**

MWB, *MHT WellBeing*; MSav, *MHT Savoring*; MCeff, *MHT Creative and executive efficiency*; MSreg, *MHT Self-regulation*; Mresil, *MHT* Resilience; Wisdom, *Wisdom and knowledge*; Human, *Humanity*; Temper, *Temperance*; STransc, *Spirituality and transcendence*;

**: *p* < 0.01.

*^a^*Column contains correlations with *WBint* after partialling out the linear effect of *WBext.*

*^b^*Column contains correlations with *WBext* after partialling out the linear effect of *WBint.*

Table 15 summarizes the correlations of the PWBSQ scales with other positivity and mental health measurement indices from Study IV. The correlation patterns are much the same as in Study I and Study II, performed with the same variables, but at a lower correlation level (see Tables 11, 13).

**TABLE 15:** Pearson’s correlations of the PWBSQ scales with other positivity and mental health measurement indices from Study IV (*n* = 1,044).

Variable	*Emot*	*Psych*	*Soc*	*Spirit*	*WBint*	*WBext*	*WBint^a^*	*WBext^b^*
GMst	0.681**	0.525**	0.411**	0.382**	0.677**	0.419**	0.586**	−0.057^+^
ActH	−0.629**	−0.440**	−0.407**	−0.368**	−0.607**	−0.410**	−0.487**	−0.008
Pexp%	0.552**	0.364**	0.336**	0.308**	0.524**	0.340**	0.422**	−0.015
PhysC	0.443**	0.366**	0.326**	0.298**	0.451**	0.330**	0.325**	0.044
Health	0.494**	0.391**	0.304**	0.277**	0.495**	0.307**	0.406**	−0.035
PhysStr	0.407**	0.387**	0.332**	0.321**	0.434**	0.345**	0.290**	0.082**
POS	0.736**	0.645**	0.581**	0.545**	0.764**	0.594**	0.612**	0.176**
DFS	0.679**	0.677**	0.667**	0.615**	0.737**	0.677**	0.519**	0.366**
H1_9	0.689**	0.659**	0.585**	0.580**	0.737**	0.615**	0.555**	0.243**
H10	0.738**	0.543**	0.481**	0.447**	0.723**	0.490**	0.604**	0.015
Savfut	0.447**	0.428**	0.368**	0.428**	0.479**	0.420**	0.292**	0.153**
Savpres	0.522**	0.510**	0.444**	0.484**	0.563**	0.490**	0.363**	0.186**
Savpast	0.393**	0.336**	0.320**	0.336**	0.404**	0.347**	0.250**	0.114**
Savor	0.507**	0.476**	0.420**	0.466**	0.539**	0.468**	0.344**	0.173**

GMst, *General mental state*; ActH, *Actual happiness*; Pexp%, *Positive experience%*; PhysC, *Physical condition*; Health, *General health condition*; PhysStr, *Physical strength*; POS, *Positivity Scale*; DFS, *Diener Flourishing Scale*; H1_9, *Huppert1_9*; H10, *Huppert10* (*Global happiness*); Savfut, *Savoring via anticipation*; Savpres, *Savoring the moment*; Savpast, *Savoring via reminiscence*; Savor, *Savoring Total*.

**p < 0.01.

*^a^*Column contains correlations with *WBint* after partialling out the linear effect of *WBext.*

*^b^*Column contains correlations with *WBext* after partialling out the linear effect of *WBint.*

Across all four studies, the PWBSQ scales demonstrate strong and consistent external validity. *WBint* emerges as the dominant correlate of general mental health, positivity, and flourishing measures, while *WBext* is more strongly associated with relational, spiritual, and character-based constructs. The asymmetry in partial correlations highlights the theoretical distinction between these domains and validates the dual-spectrum structure proposed by the Personal Wellbeing Spectrum Theory.

These findings confirm that the PWBSQ not only captures a multidimensional view of personal wellbeing but also aligns meaningfully with established psychological constructs. The instrument’s ability to differentiate between internal and external domains enhances its utility for both theoretical exploration and practical assessment.

In addition to convergent validity, the external validity of the PWBSQ was examined through analyses of divergent validity. Across Studies I–IV, the PWBSQ dimensions were tested against constructs outside the hedonic–eudaimonic framework, using the VIA-H questionnaire and selected scales of the Mental Health Test (Self-regulation, Resilience). As hypothesized, correlations with these constructs were positive but consistently lower than those observed with established wellbeing components. Specifically, Studies I and III showed that PWBSQ scales (Emotional, Psychological, Social, Spiritual) correlated below 0.50 with VIA-H virtues, except for Transcendence. In Studies II–IV, correlations with MHT subscales were week, confirming the discriminant validity of the PWBSQ. These findings demonstrate that the PWBSQ captures distinct dimensions of personal wellbeing while remaining theoretically related to broader aspects of personality functioning.

### 3.8 Results with sociodemographic indicators

An examination of the relationship between the PWBSQ scales and sociodemographic indicators yielded many significant results, although these relationships are typically rather weak (see Table 16). An exception is the subjective financial status, which correlates with the *Emot* and *WBint* scales at the medium level. These two scales also correlate weakly with the number of children. Education level correlates at the weak level with all PWBSQ scales, most strongly with the *Soc* subscale.

**TABLE 16:** Spearman’s rho between PWBSQ scales and five sociodemographic variables.

Variable	Usable cases	*Emot*	*Psych*	*Soc*	*Spirit*	*WBint*	*WBext*
Education level	14893	0.132***	0.147***	0.171***	0.107***	0.145***	0.145***
Number of children	14256	0.143***	0.042***	0.067***	0.097***	0.114***	0.087***
Age	14893	0.046***	−0.058***	−0.020*	0.046***	0.011	0.016^+^
Subjective financial status	14258	0.317***	0.259***	0.235***	0.184***	0.315***	0.219***

^+^*p* < 0.10,

*p < 0.05,

***p < 0.001.

The correlations with age do not reach even the weak level of 0.1. which may also be the result of a non-linear effect of age on wellbeing. Therefore, a one-way ANOVA was also performed with categorized age (see Table 2) and PWBSQ scales as dependent variables. Although the ANOVA results were significant for all PWBSQ scales (*p* < 0.001), the η^2^ effect size values were less than 0.10. the lower threshold of interpretability, in all but one case. In the case of the *Spirit* scale, there were surprisingly small differences between the *Spirit* means of the different age groups (the highest *Spirit* levels were achieved by the 36–50 and 51–65 age groups).

Comparing male and female means via *t*-tests and Cohen’s *d* effect size measure, the results were again significant for all PWBSQ scales (*p* < 0.001), with a week advantage for women (see Table 17).

**TABLE 17:** Comparison of male and female means via Cohen’s *d* effect size measure (*n* = 14,898).

Scale:	*Emot*	*Psych*	*Soc*	*Spirit*	*WBint*	*WBext*
Mean of men	3.82	4.17	3.63	3.74	3.95	3.68
SD of men	1.19	1.14	1.25	1.31	1.11	1.22
Mean of women	4.09	4.38	3.90	4.11	4.20	4.00
SD of women	1.07	1.03	1.19	1.21	1.00	1.15
Cohen’s *d*	0.246	0.197	0.223	0.300	0.242	0.274

To assess the effects of marital status and occupation on the means of the PWBSQ scales we also performed ANOVAs. The statistical results were again significant for all scales (*p* < 0.001), but this is not surprising for such large samples. The magnitude of effect sizes can be better characterized by the η^2^ effect size values (see Table 18).

**TABLE 18:** The η^2^ effect size in the case of marital status and occupation for the PWBSQ scales.

Sociode- mographic variable	*Emot*	*Psych*	*Soc*	*Spirit*	*WBint*	*WBext*
Marital status (*n* = 14,263)	0.037	0.015	0.010	0.010	0.029	0.011
Occupation (*n* = 13,795)	0.023	0.034	0.031	0.014	0.028	0.023

^1^That item serves to operationalize Fredrickson (2009) positivity concept.

^2^In the original questionnaire of Huppert and So (2013) this is item 6.

**FIGURE 2:**
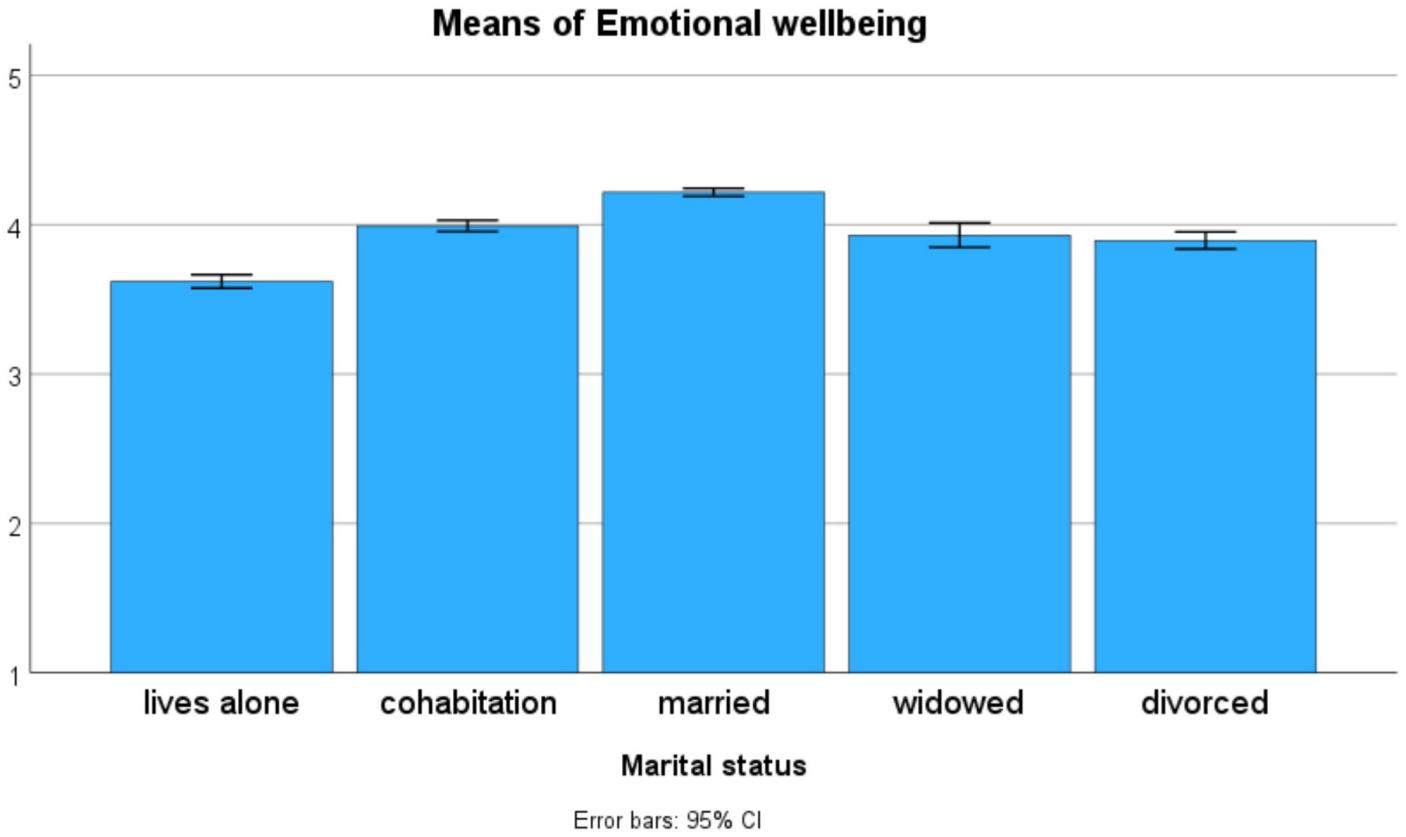
The influence of marital status on the level of emotional wellbeing (*n* = 14,263).

**FIGURE 3:**
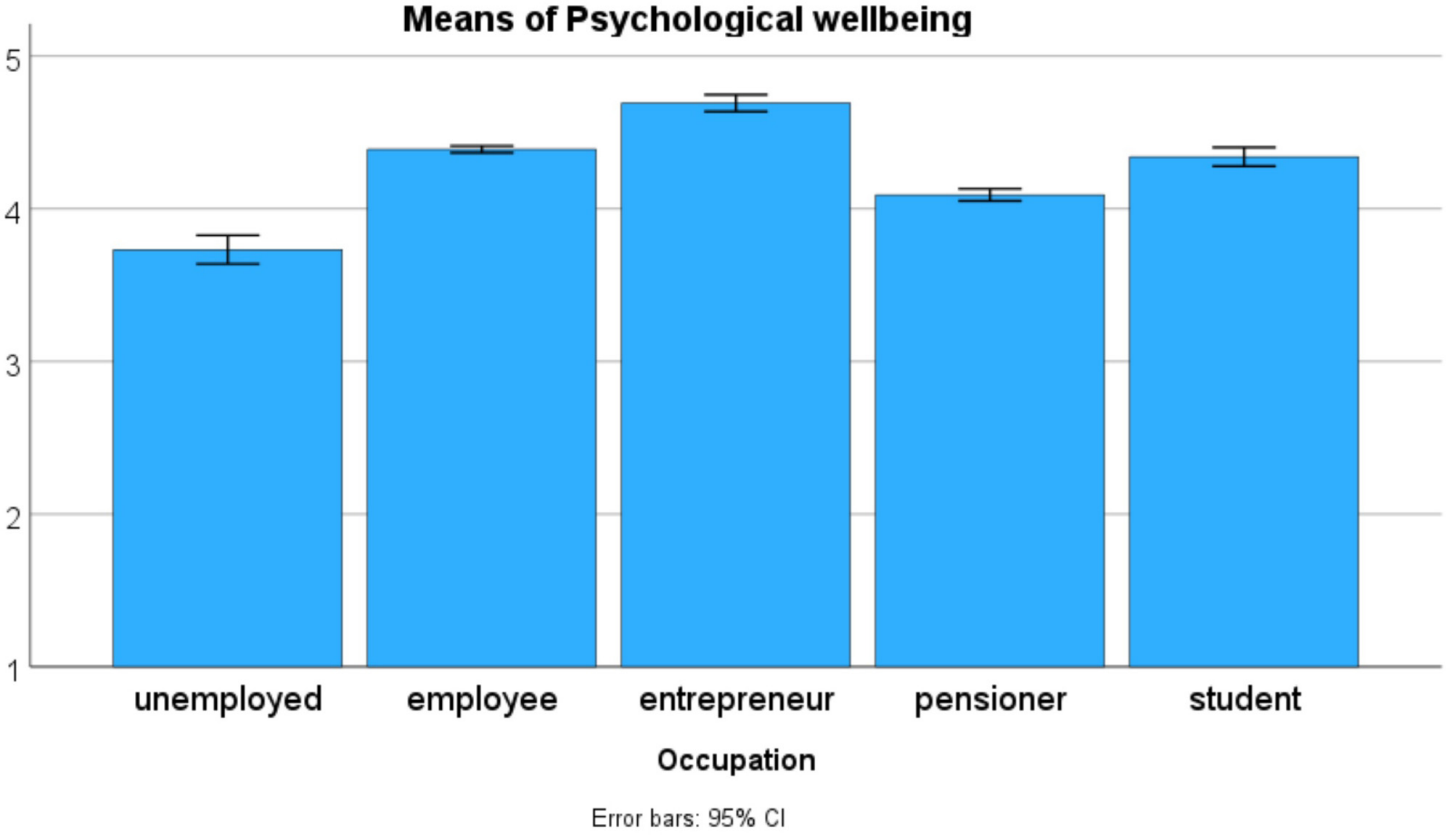
The influence of occupation on the level of psychological wellbeing (*n* = 13,795).

**FIGURE 4:**
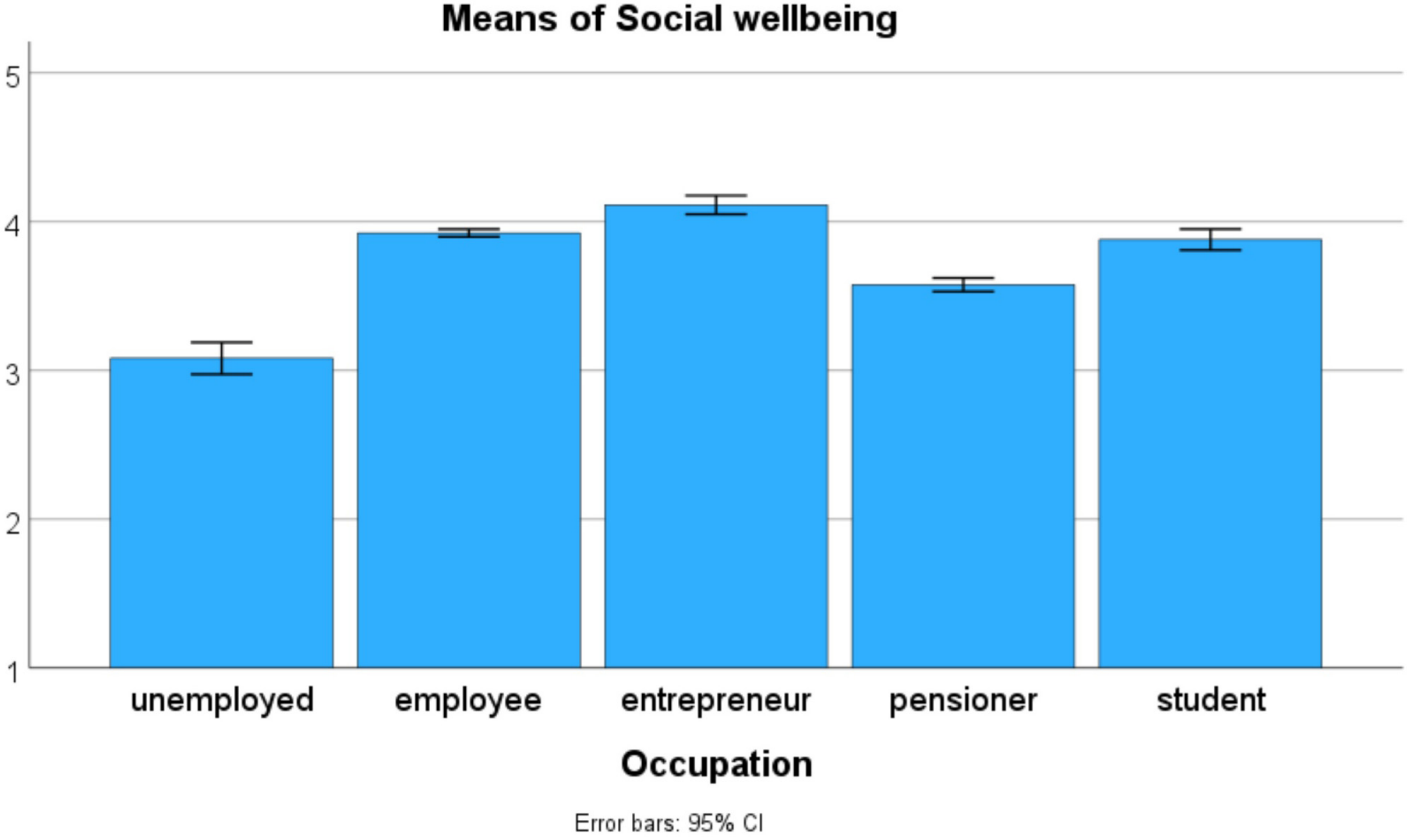
The influence of occupation on the level of social wellbeing (*n* = 13,795).

Both marital status and occupation have only small influences on the PWBSQ scales (see Table 18). In the case of marital status, the largest effect (η^2^ = 0.037) was obtained for the *Emotional wellbeing* scale, whereas in the case of occupation the largest effects were obtained for the *Psychological wellbeing* (η^2^ = 0.034) and the *Social wellbeing* (η^2^ = 0.031) scales. The influence of marital status on the level of emotional wellbeing is due to the inferior level of the *lives alone* group, and the elevated level of the *married* group (see Figure 2). The influence of occupation on the level of psychological wellbeing is due to the inferior level of the *unemployed* group, and the elevated level of the *entrepreneur* group (see Figure 3). The influence of occupation on the level of social wellbeing is again due to the inferior level of the *unemployed* group, and the elevated level of the *entrepreneur* group (see Figure 4).

## 4 Discussion

**FIGURE 5:**
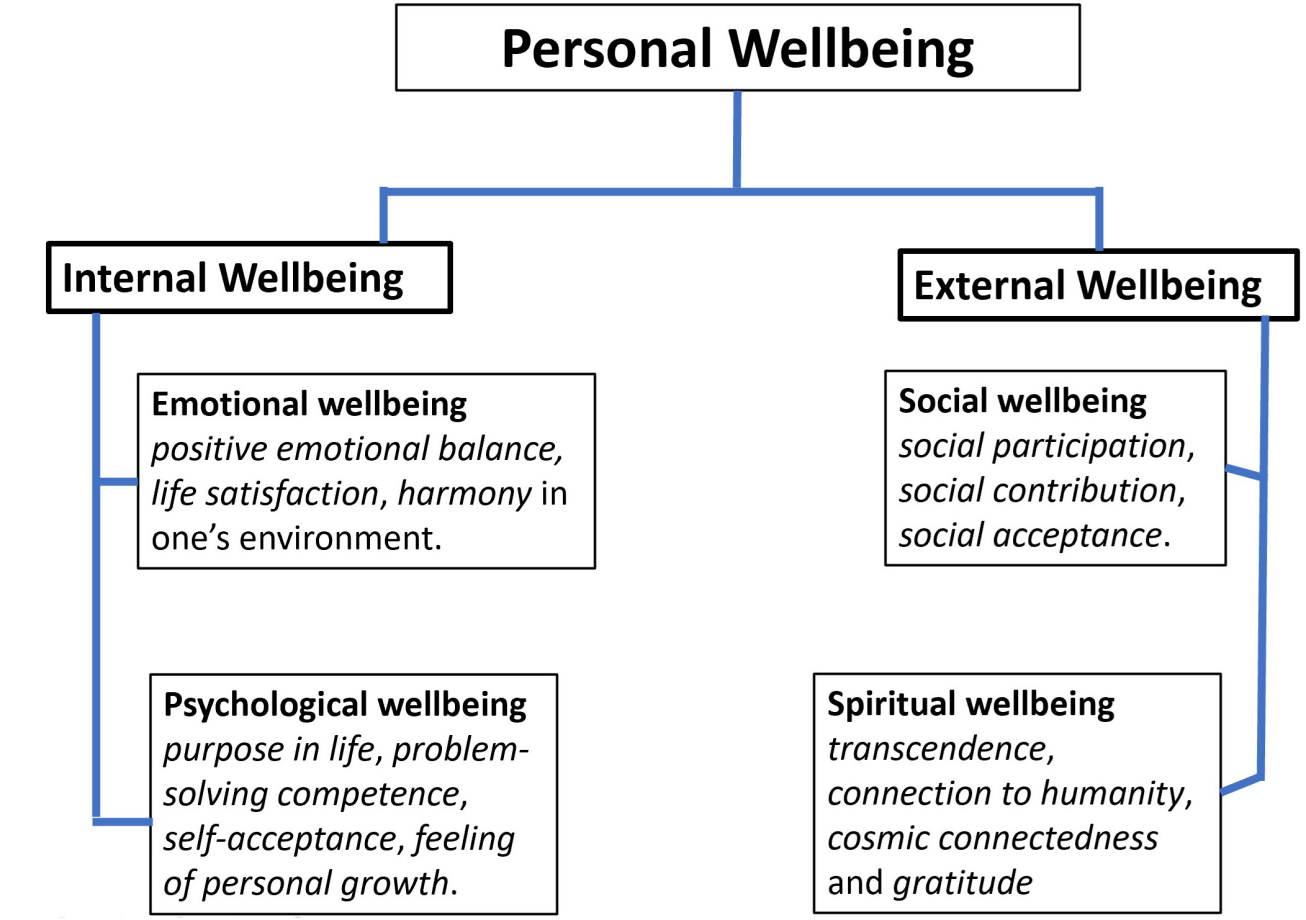
The personal wellbeing spectrum.

The objective of this paper was to conceptualize the Personal Wellbeing Spectrum Theory (PWBST) and to develop and validate the Personal Wellbeing Spectrum Questionnaire (PWBSQ) as its operationalization. PWBST offers a multidimensional framework in which emotional and psychological dimensions are organized as internal wellbeing, while social and spiritual dimensions are organized as external wellbeing. This internal–external distinction reflects two fundamental sources of human flourishing: experiences rooted in the individual’s inner world and those derived from social connections and transcendent meaning. Together, these dimensions create harmony between internal and external aspects of personal wellbeing (see Figure 5).

### 4.1 Our empirical findings provide strong support for this model

The most important finding in our online cross-sectional studies is that the second order structural validity with two second order factors (represented by *WBint* and *WBext*) and four first order factors (represented by *Emot*, *Psych*, *Soc*, and *Spirit*) of the PWBSQ was verified by CFA after removing one item from the original 17-item questionnaire, using a very large sample (Study I, *n* = 11,686; see Figure 1 and Table 3). In turn, this model with the same scales was confirmed by good fit indices in another three large (of size 1,540 in Study II, 635 in Study III, and 1,044 in Study IV) and independent samples with RMSEA values around 0.06, SRMR values around 0.04, CFI and TLI values around 0.95 (see Table 5). In addition, good internal consistency of the scales was proven (see Tables 6, 7). Configural and metric types of measurement invariance were approved for five sociodemographic variables (gender, age level, education, occupation, marital status), and also for the stringiest scalar type, but with a slight violation in the case of marital status (see Table 8). The group invariances obtained for most sociodemographic indicators confirm the stability of the explored factor model. Since Study IV was conducted among native Hungarian speakers living in the Transylvania region of Romania, the results concerning structural validity have intercultural relevance.

After the verification of structural validity, the substantive validity of PWBSQ was examined using several measures of positive psychology (Positivity Scale, Diener Flourishing Scale, Huppert Flourishing Scale, Values in Action Inventory, Mental Health Test, PERMA, as well as six questionnaire items addressing physical and mental health. Beyond structural validity, the PWBSQ showed strong substantive validity (see Tables 11–15). *WBint* correlated strongly with established hedo-eudaimonic measures such as Diener’s and Huppert’s flourishing scales, the Positivity Scale, and PERMA subscales, often with *r* > 0.70. This indicates that internal wellbeing captures the same psychological core as traditional flourishing constructs. In contrast, *WBext* showed moderate associations with these measures, highlighting that existing instruments primarily reflect internal functioning while neglecting social and spiritual domains. Divergent validity was also established, as PWBSQ scales correlated only modestly with theoretically distinct constructs such as VIA virtues and self-regulation. These findings confirm that the PWBSQ contributes unique variance by explicitly operationalizing external wellbeing alongside internal dimensions. Importantly, discriminant validity analyses confirmed that *WBext* retains unique variance: once the effect of *WBint* was partialled out, correlations with external wellbeing dropped sharply, underscoring its distinct conceptual contribution. The PWBSQ’s social wellbeing domain (*Soc*) highlights active community participation and collective belonging, offering a broader perspective than PERMA’s emphasis on close interpersonal ties. In the PWBSQ, social connectedness is operationalized as satisfaction derived from communal engagement and collective identity (e.g., *“I belong to several communities where I feel comfortable and fully accepted”; “I actively participate in communities that promote the kind of social development I support”*). By contrast, the PERMA Relationships scale defines connectedness primarily through emotional support and satisfaction with intimate relationships (e.g., *“To what extent do you feel loved?”; “How satisfied are you with your personal relationships?”*). The moderate correlations observed between these measures are consistent with theoretical expectations and underscore discriminant validity, demonstrating that the PWBSQ contributes a complementary and distinctive perspective on social wellbeing. Similarly, the spiritual wellbeing domain (*Spirit*) captures transcendence and meaning beyond physical health indicators, offering a broader conceptualization of flourishing. These distinctions were supported by moderate correlations with VIA virtues and savoring scales, which confirmed that the PWBSQ contributes complementary perspectives rather than replicating existing measures.

Sociodemographic analyses further indicated that wellbeing is shaped more by internal personal circumstances than by external demographic factors. Positive associations were found with subjective financial situation, education, and number of children, while gender differences were modest and age effects negligible. Marital status and occupation exerted only small influences, with emotional wellbeing lower among individuals living alone and psychological/social wellbeing higher among entrepreneurs. Overall, these findings suggest that personal wellbeing is determined primarily by internal functioning, with external conditions exerting weaker effects.

Taken together, the PWBSQ demonstrated strong structural, convergent, and divergent validity, while introducing two conceptual innovations: the integration of spirituality as a validated dimension of human functioning and the structural distinction between internal and external wellbeing. These features expand the theoretical landscape, provide a more differentiated understanding of flourishing, and offer a practical tool for personalized interventions.

Beyond its methodological contributions, the present study also carries important global implications. By integrating emotional, psychological, social, and spiritual dimensions into a coherent framework, the PWBSQ aligns with international priorities such as the United Nations Sustainable Development Goals, which emphasize health, wellbeing, and social inclusion across diverse populations. Its multidimensional architecture enables cross-cultural comparisons and supports the development of tailored interventions that respect cultural and contextual differences. In this way, the PWBSQ not only advances theoretical models of wellbeing but also provides a practical tool for policymakers, organizations, and practitioners seeking to promote human flourishing in varied global contexts.

## 5 Limitations

Despite its strengths, several limitations should be acknowledged. All data were self-reported, which may have introduced response biases. Although measurement invariance was confirmed across key sociodemographic groups, the samples were limited to Hungarian-speaking populations, constraining global generalizability. Convenience sampling, despite large sample sizes, may have introduced response and selection biases, and the samples cannot be considered representative even of the Hungarian population. The cross-sectional design restricts conclusions about temporal stability and developmental trajectories of internal and external wellbeing. Finally, while the inclusion of spirituality and the internal–external distinction represents a conceptual innovation, further research is needed to examine their relevance across different populations and life stages.

### 5.1 Future directions

Future studies should therefore employ larger and more diverse samples, incorporate longitudinal and cross-cultural designs, and explore the predictive utility of individual-specific wellbeing patterns for health, resilience, and intervention outcomes. Additionally, refinement of items and integration with digital assessment platforms could enhance accessibility and ecological validity. By addressing these limitations, future research will strengthen both theoretical robustness and the practical applicability of the PWBST framework and its operationalization through the PWBSQ.

Ultimately, the originality of the PWBSQ lies in bridging paradigms and offering a unifying measure that can inform both scientific inquiry and applied strategies worldwide. By capturing individual-specific patterns and distinguishing internal from external dimensions, the instrument establishes a new standard for multidimensional wellbeing assessment and opens pathways for innovative, evidence-based interventions.

## Data Availability

The research data analyzed in this paper are available from the corresponding author on request without undue reservation.

## References

[B1] Alavi M. Visentin D. C. Thapa D. K. Hunt G. E. Watson R. Cleary M. (2020). Chi-square for model fit in confirmatory factor analysis. *J. Adv. Nurs.* 76 2209–2211. 10.1111/jan.14399 32323338

[B2] Arslan G. Yıldırım M. Zangeneh M. (2022). Social inclusion and youth mental health: Evidence from cross-cultural studies. *J. Commun. Psychol.* 50 1234–1245. 10.1002/jcop.22759 34837711

[B3] Astivia O. L. O. Zumbo B. D. (2017). Population models and simulation methods: The case of the Spearman rank correlation. *Br. J. Math. Stat. Psychol.* 70 347–367. 10.1111/bmsp.12085 28140458

[B4] Beribisky N. Hancock G. R. (2023). Comparing RMSEA-based indices for assessing measurement invariance in confirmatory factor models. *Educ. Psychol. Meas.* 84 716–735. 10.1177/00131644231202949 39055094 PMC11268388

[B5] Bowen N. K. Guo S. (2012). *Structural equation modeling. Pocket guide to social work research methods.* Oxford: Oxford University Press.

[B6] Butler J. Kern M. L. (2016). The PERMA-Profiler: A brief multidimensional measure of flourishing. *Int. J. Wellbeing* 6 1–48. 10.5502/ijw.v6i3.526 32285354

[B7] Byrne B. M. (2016). *Structural equation modeling with AMOS: Basic concepts, applications, and programming*, 3rd Edn. Milton Park: Routledge, 10.4324/9781315757421

[B8] Caprara G. V. Alessandri G. Eisenberg N. Kupfer A. Steca P. Caprara M. G. (2012). The positivity scale. *Psychol. Assess.* 24 701–712. 10.1037/a0026681 22250591

[B9] Caprara G. V. Eisenberg N. Alessandri G. (2017). Positivity: The dispositional basis of happiness. *J. Happ. Stud.* 18 353–371. 10.1007/s10902-016-9728-y

[B10] Chen F. F. (2007). Sensitivity of goodness of fit indexes to lack of measurement invariance. *Struct. Equ. Model. Multidisciplinary J.* 14 464–504. 10.1080/10705510701301834

[B11] Cheung G. W. Rensvold R. B. (2002). Evaluating goodness-of-fit indexes for testing measurement invariance. *Struct. Equ. Model.* 9 233–255. 10.1207/S15328007SEM0902_5

[B12] Chirico F. Batra K. Batra R. Öztekın G. G. Ferrari G. Crescenzo P. (2023). Spiritual well-being and burnout syndrome in healthcare: A systematic review. *J. Health Soc. Sci.* 8 13–32. 10.19204/2023/sprt2

[B13] Cohen J. (1988). *Statistical power analysis for the behavioral sciences*, 2nd Edn. New Jersey: Lawrence Erlbaum.

[B14] Cohen J. (1992). Quantitative methods in psychology: A power primer. *Psychol. Bull.* 112 155–159. 10.1037//0033-2909.112.1.155 19565683

[B15] Cummins R. A. Eckersley R. Pallant J. Van Vugt J. Misajon R. (2003). Developing a national index of subjective wellbeing: The Australian unity wellbeing index. *Soc. Indicators Res.* 64 159–190. 10.1023/A:1024704320683

[B16] Curran P. J. West S. G. Finch J. F. (1996). The robustness of test statistics to nonnormality and specification error in confirmatory factor analysis. *Psychol. Methods* 1 16–29. 10.1037/1082-989X.1.1.16

[B17] Deci E. L. Ryan R. M. (2012). “Self-Determination Theory,” in *Handbook of theories of social psychology*, Vol. Vol. 1 eds Van Lange P. A. M. Kruglanski A. W. Higgins E. T. (Thousand Oaks: Sage), 416–437. 10.4135/9781446249215.n21

[B18] Diener E. Emmons R. A. Larsen R. J. Griffin S. (1985). The satisfaction with life scale. *J. Pers. Assess.* 49 71–75. 10.1207/s15327752jpa4901_13 16367493

[B19] Diener E. Wirtz D. Tov W. Kim-Prieto C. Choi D. Oishi S. (2010). New measures of well-being: Flourishing and positive and negative feelings. *Soc. Indicators Res.* 39 247–266. 10.1007/s11205-009-9493-y

[B20] Dodge R. Daly A. P. Huyton J. Sanders L. D. (2012). The challenge of defining wellbeing. *Int. J. Wellbeing* 2 222–235. 10.5502/ijw.v2i3.4 32285354

[B21] Dunn T. J. Baguley T. Brunsden V. (2014). From alpha to omega: A practical solution to the pervasive problem of internal consistency estimation. *Br. J. Psychol.* 105 399–412. 10.1111/bjop.12046 24844115

[B22] Fredrickson B. (2009). *Positivity: Groundbreaking research reveals how to embrace the hidden strength of positive emotions, overcome negativity, and thrive.* New York, NY: Crown Publishers.

[B23] Furnham A. Lester D. (2012). The development of a short measure of character strength. *Eur. J. Psychol. Assess.* 28 95–101. 10.1027/1015-5759/a000096

[B24] Gana K. Broc G. (2019). *Structural equation modeling with lavaan.* Hoboken, NJ: John Wiley & Sons, Inc.

[B25] Gao C. Shi D. Maydeu-Olivares A. (2020). Estimating the maximum likelihood root mean square error of approximation (RMSEA) with non-normal data: A Monte-Carlo study. *Struct. Equ. Model. Multidisciplinary J.* 27 192–201. 10.1080/10705511.2019.1637741

[B26] Gottlieb P. (2009). *The virtue of Aristotle’s ethics.* Cambridge: Cambridge University Press.

[B27] He J. van de Vijver F. (2012). Bias and equivalence in cross-cultural research. *Online Readings Psychol. Culture* 2 10.9707/2307-0919.1111

[B28] Hu L. Bentler P. M. (1999). Cutoff criteria for fit indexes in covariance structure analysis: Conventional criteria versus new alternatives. *Struct. Equ. Modeling Multidisciplinary J.* 6 1–55. 10.1080/10705519909540118

[B29] Huppert F. A. So T. T. (2013). Flourishing across Europe: Application of a new conceptual framework for defining well-being. *Soc. Indicators Res.* 110 837–861. 10.1007/s11205-011-9966-7 23329863 PMC3545194

[B30] IBM Corp. (2020). *IBM SPSS statistics for windows (Version 27.0).* Armonk, NY: IBM Corp.

[B31] Kahneman D. Diener E. Schwarz N. (1999). *Well-being: Foundations of hedonic psychology.* New York, NY: Russell Sage Foundation.

[B32] Kelley K. (2007). *Methods for the Behavioral, Educational, and Social Sciences (MBESS).* Available online at: https://CRAN.R-project.org/package=MBESS (accessed December 16, 2025). 10.3758/bf03192993 18183915

[B33] Keyes C. L. M. (1998). Social well-being. *Soc. Psychol. Quart.* 61 121–140. 10.2307/2787065

[B34] Linton M. J. Dieppe P. Medina-Lara A. (2016). Review of 99 self-report measures for assessing well-being in adults: Exploring dimensions of well-being and development of a conceptual framework. *BMJ Open* 6:e010641. 10.1136/bmjopen-2015-010641 27388349 PMC4947747

[B35] Maslow A. H. (1968). *Toward a psychology of being*, 2nd Edn. New York, NY: Van Nostrand Reinhold.

[B36] McDonald R. P. (1999). *Test theory: A unified treatment.* New Jersey: Lawrence Erlbaum Associates Publishers.

[B37] Milfont T. L. Fischer R. (2010). Testing measurement invariance across groups: Applications in cross-cultural research. *Int. J. Psychol. Res.* 3 111–121. 10.21500/20112084.857

[B38] Momin M. M. Rolla K. P. (2024a). Exploring the multi-faceted nature of wellbeing across genders: Evaluating the antecedence of psychological capital and life satisfaction. *Gender Issues* 41:11. 10.1007/s12147-024-09328-6

[B39] Momin M. M. Rolla K. P. (2024b). Alliance or association? Exploring the effect of work–family balance on workplace well-being, and the mediating effect of work attitudes. *IIM Kozhikode Soc. Manag. Rev.* 10.1177/22779752241242247

[B40] Momin M. M. Rolla K. P. (2024c). Juggling life and work: Unravelling the moderated-mediation effect of work engagement and turnover intention. *Industrial Commercial Train.* 56 359–376. 10.1108/ICT-07-2023-0045

[B41] Muthén L. K. Muthén B. O. (1998–2011). *Mplus user’s guide*, 6th Edn. Los Angelea, CA: Muthén & Muthén.

[B42] Nagy H. Magyaródi T. Vargha A. Oláh A. (2022). The development of a shortened hungarian version of the savoring beliefs inventory. *Mentálhigiéné Pszichoszomatika* 23 95–111. 10.1556/0406.23.2022.003

[B43] Oláh A. (2021). *A Globális Jól-lét Modell kidolgozása és empirikus validitásának igazolása a személyiségtényezők figyelembe vételével. A K1A: 116965 számú kutatás zárójelentése. [Developing the Global Well-being Model and demonstrating its empirical validity by taking into account personality factors. Final report of research K1A: 116965].* Hungarian. Available online at: https://www.otka-palyazat.hu/download.php?type=zarobeszamolo&projektid=116965 (accessed January 25, 2025)

[B44] Oláh A. Vargha A. Caprara G. V. (2025). Hungarian validation of the positivity scale. *Mentálhigiéné Pszichoszomatika* 26 1–14. 10.1556/0406.2025.00088

[B45] Peterson C. Seligman M. E. (2004). *Character strengths and virtues: A handbook and classification*, Vol. 1. Oxford: Oxford University Press.

[B46] Putnick D. L. Bornstein M. H. (2016). Measurement invariance conventions and reporting: The state of the art and future directions for psychological research. *Dev. Rev.* 41 71–90. 10.1016/j.dr.2016.06.004 27942093 PMC5145197

[B47] Rosseel Y. (2012). lavaan: An R Package for structural equation modeling. *J. Stat. Softw.* 48 1–36. 10.18637/jss.v048.i02

[B48] Ryff C. D. (1989). Happiness is everything, or is it? Explorations on the meaning of psychological well-being. *J. Pers. Soc. Psychol.* 57 1069–1081. 10.1037/0022-3514.57.6.1069

[B49] Ryff C. D. (2021). Spirituality and well-being: Theory, science, and the nature connection. *Religions* 12:914. 10.3390/rel12110914 34881052 PMC8651234

[B50] Seligman M. (2018). PERMA and the building blocks of well-being. *J. Positive Psychol.* 13 333–335. 10.1080/17439760.2018.143746

[B51] Seligman M. E. (2011). *Flourish: A visionary new understanding of happiness and well-being.* New York, NY: Simon and Schuster.

[B52] Tennant R. Hiller L. Fishwick R. Platt S. Joseph S. Weich S. (2007). The Warwick–edinburgh mental well-being scale (WEMWBS): Development and UK validation. *Health Quality Life Outcomes* 5:63. 10.1186/1477-7525-5-63 18042300 PMC2222612

[B53] van de Schoot R. Lugtig P. Hox J. (2012). A checklist for testing measurement invariance. *Eur. J. Dev. Psychol.* 9 486–492. 10.1080/17405629.2012.686740

[B54] Varga B. A. Oláh A. Vargha A. (2022). A PERMA-Profiler Kérdőív magyar nyelvű megbízhatóságának és érvényességének a vizsgálata [Testing the reliability and validity of the PERMA-Profiler Questionnaire in Hungarian]. *Mentálhigiéné Pszichoszomatika* 23 33–64. Hungarian. 10.1556/0406.23.2022.001

[B55] Varga B. A. Oláh A. Urbán R. Jakab J. Vargha A. (2025). Assessing levels of Flourishing: Diener’s Flourishing Scale reveals greater insights into low flourishing in a Hungarian sample. *Curr. Psychol.* 44 3406–3419. 10.1007/s12144-025-07356-3

[B56] Vargha A. Bánsági P. (2022). ROP-R: A free multivariate statistical software that runs R packages in a ROPstat framework. *Hungarian Stat. Rev.* 5 3–29. 10.35618/HSR2022.02.en003

[B57] Vargha A. Torma B. Bergman L. R. (2015). ROPstat: A general statistical package useful for conducting person-oriented analyses. *J. Person Oriented Res.* 1 87–98. 10.17505/jpor.2015.09

[B58] Vargha A. Zábó V. Török R. Oláh A. (2020). A jóllét és a mentális egészség mérése: a Mentális Egészség Teszt. [Measuring well-being and mental health: the Mental Health Test]. *Mentálhigiéné Pszichoszomatika* 21 281–322. Hungarian. 10.1556/0406.21.2020.014

[B59] Yildirim M. Alshehri N. A. (2019). Does self-esteem mediate the relationship between gratitude and subjective well-being? *Polish Psychol. Bull.* 50 149–156. 10.24425/ppb.2019.126030

[B60] Zábó V. Oláh A. Vargha A. (2022). A new complex mental health test in positive psychological framework. *Front. Psychol.* 13:775622. 10.3389/fpsyg.2022.775622 36118505 PMC9479003

[B61] Zábó V. Oláh A. Erát D. Vargha A. (2023). Assessing your strengths – Hungarian validation of the 24-Item Values in Action Inventory of Strengths on a large sample. *Eur. J Mental Health* 18:e0012. 10.5708/EJMH.18.2023.0012

[B62] Zhang W. Balloo K. Hosein A. Medland E. (2024). A scoping review of well-being measures: Conceptualisation and scales for overall well-being. *BMC Psychol.* 12:585. 10.1186/s40359-024-02074-0 39443963 PMC11515516

