# Supplementary material for: The spectral model of personal wellbeing and the validity of the Personal Wellbeing Spectral Questionnaire
Source: Front Psychol. 2026 Jan 15;16:1719574. doi: 10.3389/fpsyg.2025.1719574 (PMC12852471; doi:10.3389/fpsyg.2025.1719574)
Supplement: Supplementary file 1 [file Data_Sheet_1.docx]

**Appendix**

**The Personal Well-being Spectrum Questionnaire (PWBSQ) [A Perszonális Jóllét Kérdőív]**

The following statements are designed to provide information about your perceptions of wellness. Please consider each statement carefully and thoughtfully, then enter an X to indicate the response option with which you most agree. There are no right or wrong answers. [Az alábbi állítások azt írják le, hogy valaki hogyan gondolkodhat Önmagáról. Kérjük, jelezze, hogy mennyiben ért egyet Önmagára vonatkoztatva ezekkel az állításokkal!]

|  | Strongly disagree [Egyáltalán nem értek egyet] | Disagree [Részben egyetértek] | Slightly disagree [Inkább nem értek egyet] | Slightly agree [Inkább egyetértek] | Agree  [Nagyon egyetértek] | Strongly agree  [Teljesen egyetértek] |
| --- | --- | --- | --- | --- | --- | --- |
| i01emo - I consider myself a generally happy person. [Alapvetően én egy boldog ember vagyok] |  |  |  |  |  |  |
| i02emo - In my daily life, joyful moments occur at least three times more often than sorrowful ones. [Az én mindennapjaimban legalább háromszor több az öröm, mint a bánat] |  |  |  |  |  |  |
| i03emo - I am satisfied with nearly all aspects of my life. [Életemmel szinte minden vonatkozásban elégedett vagyok] |  |  |  |  |  |  |
| i04emo - My immediate and broader environment is characterized by harmony. [Szűkebb és tágabb világomat is a harmónia jellemzi] |  |  |  |  |  |  |
| i05psy - My life is guided by meaningful goals. [Az életem tele van értelmes célokkal] |  |  |  |  |  |  |
| i06psy - I manage problems effectively in both my work and personal life. [Jól boldogulok, amikor a magánéletemben vagy a munkám során adódó feladatokat kell megoldanom] |  |  |  |  |  |  |
| i07psy - I experience continuous personal development year after year. [Évről-évre folyamatosan fejlődöm, szinte minden téren] |  |  |  |  |  |  |
| i08emo - I am satisfied with what I have achieved in life so far. [Elégedett vagyok azzal, amit eddig az életemben sikerült elérnem] |  |  |  |  |  |  |
| i09soc - I belong to several communities where I feel comfortable and fully accepted. [Sok olyan közösséghez tartozom, ahol jól érzem magam és ahol szívesen fogadnak] |  |  |  |  |  |  |
| i10spi - I feel joy in being part of a larger community committed to shaping the future responsibly. [Az érzés, hogy része lehetek az emberiség jövőjéért felelősséget viselők nagy közösségének, boldogsággal tölt el] |  |  |  |  |  |  |
| i11soc - I actively participate in communities that promote the kind of social development I support. [Olyan közösségek tagjaként működöm aktívan, akik a társadalom fejlesztésének azon irányát szorgalmazzák, amellyel én is egyetértek] |  |  |  |  |  |  |
| i12soc - I live among communities that are accepting and supportive. [Elfogadó és támogató közösségekben élem az életemet] |  |  |  |  |  |  |
| i13spi - I actively support efforts to strengthen unity among people for a sustainable future. [Az emberek összefogásának erősítése az élhetőbb jövő érdekében olyan cél, amelyért magam is aktívan teszek] |  |  |  |  |  |  |
| i14spi - I am energized by the moments when I can feel that I am part of the universe. [Sok energiával töltenek fel azok a pillanatok, amikor átélhetem, hogy magam is része vagyok a világegyetemnek] |  |  |  |  |  |  |
| i15psy - I am satisfied with myself in nearly every respect. [Szinte minden téren elégedett vagyok magammal] |  |  |  |  |  |  |
| i16soc - Through my work and social engagement, I clearly contribute to shaping my social environment. [Munkámmal és közösségi aktivitásommal egyértelműen fejlesztője vagyok társas világomnak] |  |  |  |  |  |  |
| i17spi - I feel grateful knowing that many people in the world share my values, and I feel a sense of belonging with them, even if we’ve never met. [Hálás vagyok a sorsnak, hogy nagyon sok hozzám hasonlóan gondolkodó ember él a Földön, akikkel ismeretlenül is egy közösségbe tartozónak érzem magam] |  |  |  |  |  |  |
